# Engineering a dimeric single-domain antibody for improved detection and neutralization of amyloid-β oligomers

**DOI:** 10.1038/s42003-026-09740-6

**Published:** 2026-03-27

**Authors:** Liliana Napolitano, Devkee M. Vadukul, Alessandra Bigi, Pasquale Palladino, Cristina Cecchi, Fabrizio Chiti, Roberta Cascella, Francesco A. Aprile

**Affiliations:** 1 Department of Experimental and Clinical Biomedical Sciences, Section of Biochemistry, University of Florence, Florence, Italy; 2 Department of Chemistry, Molecular Sciences Research Hub, Imperial College London, London, UK; 3 Dipartimento di Chimica ‘Ugo Schiff’–DICUS, University of Florence, Florence, Italy; 4 Institute of Chemical Biology, Molecular Sciences Research Hub, Imperial College London, London, UK; 5 Present Address: Institute of Applied Physics “Nello Carrara”, Italian National Research Council, Sesto Fiorentino, Italy

**Keywords:** Biochemistry, Biophysics

## Abstract

Soluble Aβ oligomers are regarded as major neurotoxic agents in Alzheimer’s disease. Several monoclonal antibodies have been developed to target Aβ oligomers, but most of them show limited specificity binding also to monomers and fibrils. To generate an antibody with high specificity for the oligomers, we aimed to increase the efficiency and sensitivity of a human VH–derived Aβ-oligomer-specific single domain antibody, called DesAb-O. We engineered a dimeric DesAb-O variant, DiDesAb-O, which showed significantly higher binding affinity for Aβ oligomers as compared to the monomeric sdAb. DiDesAb-O selectively detected Aβ_42_ oligomers not only in vitro and in cultured cells using synthetic preparations, but also in the cerebrospinal fluid from Alzheimer’s patients. Moreover, it inhibited the binding of these toxic species to cellular membranes and neutralized their neurotoxicity both in cells and in patient-derived cerebrospinal fluid at lower concentrations compared to DesAb-O. These results indicate that rational dimerization of single-domain antibodies can substantially enhance target engagement and functional efficacy, providing a promising strategy for the development of improved diagnostic and therapeutic molecules for Alzheimer’s disease.

## Abstract (web summary)

Development of a dimeric single-domain antibody against amyloid-β oligomers reveals markedly enhanced affinity and conformational selectivity in vitro and in biological samples compared with the monomeric antibody fragment.

Approximately 55 million people worldwide suffer from dementia, with Alzheimer’s disease (AD) being the leading cause, accounting for an estimated 60–80% of cases^1^. This number is predicted to double by 2050^1^, thus, the need for accurate and early diagnostic tools has become increasingly urgent.

AD is primarily characterized by brain atrophy and synapse loss associated with the accumulation of extracellular amyloid-beta (Aβ) plaques and intracellular tau neurofibrillary tangles^2,3^. Notably, Aβ aggregation begins at least two decades before the appearance of clinical symptoms of AD^4–6^. Aβ exists in the form of peptides of varying length, ranging from 37 to 49 residues^7^. The deposition of amyloid fibrils of the 42-residue Aβ (Aβ_42_) in specific brain areas as neuritic plaques represents a major pathological hallmark of AD^8^, and an early histopathological feature revealed by the earliest biomarkers, such as amyloid plaques by positron emission tomography (PET) or Aβ_42_ depletion from the cerebrospinal fluid (CSF)^4^. These features precede the clinical diagnosis of dementia by ca. 5–15 years^6^.

While Aβ_42_ fibrils are a pathological feature of AD, growing evidence suggests that it is the smaller, soluble aggregation intermediates, i.e., the oligomers, that are primarily responsible for the neurotoxic effects observed in AD^2,9–12^. Increased oligomer levels have been detected in the brain and CSF of AD patients compared to age-matched controls^9,13–17^ and have shown to correlate stronger with the severity of cognitive decline and with the loss of synaptic markers compared to mature amyloid fibrils^11–18^. Despite the prevalence of oligomers in AD, suitable sensitive methods for their routine and reliable detection, quantification and isolation from biological fluids have been lacking due to their transient and heterogeneous nature.

Recent progress in the development of conformation-specific monoclonal antibodies (mAbs) has enabled the targeting of toxic Aβ_42_ oligomers^9,13,14,16,17,19^. Additionally, mAbs have been developed to target Aβ in AD patients as a therapeutic strategy^20^, with several currently undergoing clinical trials^21,22^. Among them, aducanumab^23,24^, lecanemab^22,25^ and donanemab^26^ have received FDA approval for AD treatment, although production of the former has been later discontinued by its producer company, Biogen. Use of these mAbs in clinical practice has been associated with adverse effects, including intracerebral hemorrhages and amyloid-related imaging abnormalities^25–27^.

The discovery of conformation-sensitive antibodies (Abs) and the approval of monoclonal antibodies (mAbs) as the first disease-modifying therapy for AD have been significant breakthroughs. However, their severe side effects represent a significant limitation for clinical application. On the contrary, single-domain Abs (sdAbs), formed only by the variable fragment of the heavy chain of camelid antibodies, are emerging as promising candidates for AD diagnosis and therapy, due to their small size, high solubility and low immunogenicity^28–31^. Previously published work demonstrated that targeting the region 29–36 of Aβ42 with rationally designed human VH–derived sdAbs based on the VH framework of the clinically approved mAb Trastuzumab^32,33^, called DesAbs^34^, can interfere with the Aβ aggregation process^35^. Among them, DesAb-O was found to preferentially bind to Aβ oligomers rather than the monomers or fibrils, as this region is likely to be solvent-exposed when the peptide is oligomeric, before becoming buried in mature fibrils^36^. In a further study, DesAb-O was found to be able to selectively detect synthetic Aβ_42_ oligomers both in vitro and in cultured cells, and to prevent their associated neuronal dysfunction^37^. Notably, DesAb-O could also identify and neutralize Aβ_42_ oligomers present in the CSF samples (CSFs) of AD patients with respect to healthy individuals^37^.

Recent studies have shown that multivalency is an effective strategy to enhance the effects of sdAbs, leading to improved protein detection, increased avidity and binding ability for protein aggregates^38,39^. Building on this concept, we engineered a dimeric variant of DesAb-O, named DiDesAb-O, to further enhance its binding properties and refine its ability to detect and neutralize oligomers. Our data show that DiDesAb-O has an enhanced anti-aggregation activity and sensitivity to detect Aβ_42_ oligomers in vitro and in cultured cells as compared to the monomeric counterpart DesAb-O, and is able to detect and neutralize toxic species in both synthetic Aβ_42_ samples and AD CSF at lower concentrations as compared to DesAb-O. Our findings also demonstrate that the rational engineering of dimeric sdAbs is a promising avenue for developing potential diagnostic and therapeutic molecules targeting AD and other neurodegenerative diseases.

## Results

### DiDesAb-O design strategy

**Fig. 1:**
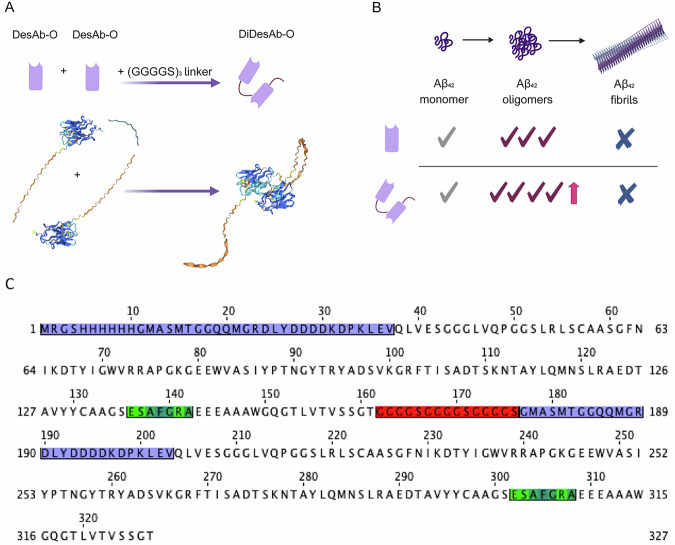
DiDesAb-O design and amino acidic residue sequence. **A** Schematic representation of the strategy used to obtain DiDesAb-O. At the bottom, the AlphaFold 3^73^ predictions of the protein variants are shown: on the left, two separate DesAb-O monomers and the (GGGGS)_3_ flexible linker (blue); on the right, the predicted DiDesAb-O structure. **B** Schematic representation of the aim of the study. We designed a dimeric structure of DesAb-O to obtain enhanced sensitivity and binding avidity for Aβ_42_ oligomers, while avoiding increased binding to Aβ_42_ monomers or fibrils. Panels **A** and **B** were created in https://BioRender.com. **C** DiDesAb-O amino acid residue sequence. N-termini are highlighted in light purple, the flexible linker (GGGGS)_3_ is highlighted in red, whereas the antibody binding sites are highlighted in shades of green. The sequence was obtained with the Jalview software (version 2.11.40).

The rationale behind the engineering of DiDesAb-O was to enhance the binding to the Aβ_42_ oligomers by increasing the avidity relative to the previously studied monomeric DesAb-O^36,37^. It is conceivable that the dimer may be capable of binding to two exposed epitopes either within the same oligomeric assembly or on separate oligomers. This could result in an enhanced binding avidity for Aβ_42_ oligomers. To obtain DiDesAb-O, the C-terminus of one DesAb-O monomer was linked to the N-terminus of another DesAb-O monomer via a flexible linker, consisting of three repeats of the Gly-Gly-Gly-Gly-Ser sequence, i.e., (GGGGS)_3_^40^ as illustrated in Fig. 1A. This design ensures that DiDesAb-O represents a near two-fold replication of the original monomer. All sequence elements of DesAb-O are retained, except that the His-tag is present only on the first monomer and absent from the second. Notably, DesAb-O N-terminus contains residues that are preferred in protein linker regions^40^, allowing it to function as an extension of the linker itself and effectively increasing the overall linker length (Fig. 1A). This extended linker enhances the flexibility and ability of DiDesAb-O to bind distant epitopes, such as those found in the Aβ_42_ oligomers^36,37^, consistent with the overall aim of the design (schematically depicted in Fig. 1B). The entire amino acid sequence of DiDesAb-O is shown in Fig. 1C.

### Structural characterization of DiDesAb-O

DiDesAb-O was expressed in *E. coli* and purified, as detailed in the “Materials and Methods” section and in Supplementary Fig 1. The molecular weight of the purified construct, its secondary structure and thermal stability were characterized by far-UV circular dichroism (CD) and electrospray ionization mass spectrometry (ESI-MS), as reported in Supplementary Fig. 2A–D. Far-UV CD spectroscopy showed that DiDesAb-O exhibits a predominant β-sheet secondary structure, similar to that observed for the monomer (Supplementary Fig. 2A). To provide a quantitative interpretation of the far-UV CD spectrum of DiDesAb-O and compare it with that previously obtained for DesAb-O^36^, the BestSel software^41^

(v1.3.230210) was employed (Supplementary Table 1). BestSel analysis yielded similar proportions of antiparallel β-sheet secondary structure for DiDesAb-O compared to DesAb-O (61% and 57%, respectively). Notably, DesAb-O was found to comprise 7% parallel β-sheet, an element that is entirely absent from the dimeric structure profile. Overall, the total β-sheet structure is similar for dimeric and monomeric sdAbs (61% and 64%, respectively). Conversely, the dimeric sdAb was found to exhibit a 3% α-helix, possibly attributable to the presence of the linker, which is absent for DesAb-O. The quantification of turns plus disordered secondary structure is similar in the two cases, amounting to 36%. Based on the ESI-MS spectrum, the mass of the purified protein was determined to be 34,270 Da (Supplementary Fig. 2B). This experimental value closely matched the expected mass of DiDesAb-O, calculated as 34,269 Da using the ExPASy ProtParam tool^42^.

To assess whether DiDesAb-O had similar structural stability to DesAb-O, we performed a thermal denaturation experiment to determine the temperature of half-denaturation (*T*_*m*_) (Supplementary Fig. 2C, D). CD spectra were recorded between 20 and 90 °C. The mean molar residue ellipticity per residue ([*θ*]_res_) at 210 nm was extracted from each CD spectrum and plotted as a function of temperature. The data were converted into fraction folded (%) values and fitted with a two-state model. The two sdAbs fragments had similar *T*_*m*_ values (73.7 °C for DiDesAb-O and 74.6 °C for DesAb-O). The slight decrease of *T*_*m*_ value and cooperativity for DiDesAb-O relative to DesAb-O likely results from the high net charge of DesAb-O monomers that generates a further repulsion between the two monomeric units in DiDesAb-O, with consequent destablization (lower *T*_*m*_ value) and lower enthalpy change at the *T*_*m*_ (Δ*H*_*m*_), resulting in lower transition cooperativity.

The binding affinity of DiDesAb-O for Aβ-derived diffusible ligands (ADDLs)^43^ was determined using surface plasmon resonance (SPR) analysis and compared with that measured for DesAb-O. ADDLs were covalently immobilized on the carboxymethylated dextran surface of flow cell 2 (FC2) of a gold sensor chip, reaching ~1000 response units (RU), according to a previously reported procedure^44,45^. Flow cell 1 (FC1) was used as the reference surface. Twofold serial dilutions of DiDesAb-O or DesAb-O (analytes) were freshly prepared in PBS (pH 7.4) running buffer and injected over the immobilized ADDLs at 37 °C using the single-cycle kinetics (SCK) method. All experiments were performed in triplicate, and the results are shown in Supplementary Fig. 2E, while raw SPR sensograms are reported in Supplementary Fig. 3 and Supplementary Table 2. Fitting of the SPR binding curves for DiDesAb-O (Supplementary Fig. 2E, left) yielded an apparent dissociation constant (*K*_*D*_) of 5.0 ± 1.3 nM (*R*² = 0.9816). In contrast, under the same conditions, DesAb-O produced signals too weak to allow a reliable determination of the *K*_*D*_ within the tested concentration range. However, injections of DesAb-O at micromolar concentrations (Supplementary Fig. 2E, right) enabled the estimation of a *K*_*D*_ of 4.9 ± 3.6 μM (*R*² = 0.9923). This affinity is lower (i.e., higher *K*_*D*_) than the ±0.4 μM value previously reported using solution-phase isothermal calorimetry (ITC)^36^. The discrepancy likely reflects differences between the heterogeneous-phase SPR format (ADDLs covalently immobilized; sdAbs in solution) and homogeneous ITC measurements, where *K*_*D*_ can diverge. Nevertheless, in this SPR assay, DiDesAb-O showed clear nanomolar responses with an apparent *K*_*D*_ extremely lower than DesAb-O. Together with structural analyses, these results show that engineering DesAb-O into a dimeric form via a flexible linker generated a stable sdAb dimer that retain secondary structure and thermal stability similar to its monomeric counterpart, while achieving stronger binding affinity.

### DiDesAb-O slows down Aβ_42_ aggregation more effectively than DesAb-O

**Fig. 2:**
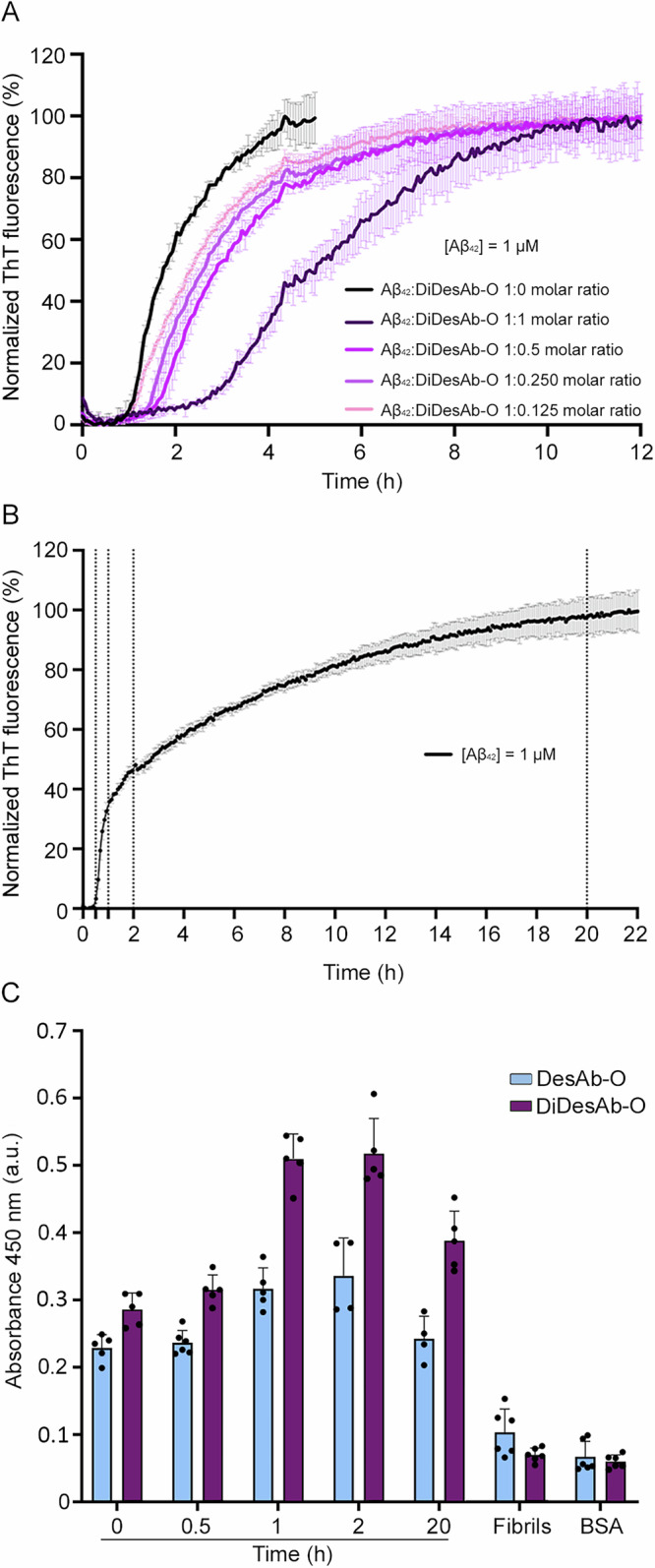
DiDesAb-O interferes with the Aβ_42_ aggregation process and has improved binding properties for Aβ_42_ oligomers. **A** Representative ThT fluorescence assays of 1 μM Aβ_42_ at the indicated Aβ_42_:DiDesAb-O molar ratios: 1:0 (black), 1:1 (dark purple), 1:0.5 (bright purple), 1:0.25 (dark pink) and 1:0.125 (pink). **B** ThT-based in vitro aggregation assay of 1 μM Aβ_42_ in the absence of DiDesAb-O (average of three replicates is shown). The black dashed lines indicate the timepoints at which samples were collected from the aggregation reaction to perform the ELISA experiment. In both panels, error bars refer to standard deviations. **C** ELISA experiment performed on samples collected from the aggregation reaction shown in (**B**), using DesAb-O and DiDesAb-O as primary Abs. Aβ_42_ fibrils obtained after 4 days of incubation at 37 °C were used as a control. BSA signals represent the background absorbance values. The bar corresponding to DesAb-O is colored light blue, while the one corresponding to DiDesAb-O is purple. Data are expressed as fold change in absorbance relative to the DesAb-O absorbance at the initial timepoint (*t* = 0’). Error bars indicate S.D. calculated from *n* = 4–6 internal replicates.

To assess the ability of DiDesAb-O to interfere with Aβ_42_ aggregation, we performed Thioflavin T (ThT) assays on solutions containing 1 μM monomeric Aβ_42_ in the presence of various Aβ_42_:DiDesAb-O molar ratios (1:1, 1:0.5, 1:0.25, and 1:0.125), as reported in Fig. 2A and Supplementary Fig. 4A. Solutions containing only the highest DiDesAb-O concentration (1 μM) in the absence of Aβ_42_ were tested as a control (Supplementary Fig. 4A). DiDesAb-O significantly reduced the Aβ_42_ aggregation process at the 1:1 molar ratio, increasing the aggregation half-times (*t*_50_) by approximately 2-fold compared to Aβ_42_ alone (3.2 ± 1.1 and 1.5 ± 0.4 h, respectively). Furthermore, the *t*_50_ value decreased as a function of DiDesAb-O concentration (Supplementary Table 3), demonstrating the ability of DiDesAb-O to interfere with the Aβ_42_ aggregation even at sub-stoichiometric concentrations (Fig. 2A). *t*_50_ values were determined as the mean of three independent experiments (Supplementary Table 3), whereas Fig. 2A and Supplementary Fig. 4A only show one representative kinetic trace per condition.

To evaluate the effect of DiDesAb-O with respect to DesAb-O, we tested increasing molar ratios of Aβ_42_:DesAb-O (1:1, 1:2) and compared the kinetic traces with those obtained with DiDesAb-O, as represented in Supplementary Fig. 4A, B. The aggregation profiles obtained with increasing DesAb-O molar ratios slightly increased the *t*_*50*_ value (1.8 ± 0.6 h for 1:2, 1.7 ± 0.4 h for 1:1, 1.5 ± 0.4 h for 1:0 Aβ_42_:DesAb-O, respectively), slowing down the Aβ_42_ aggregation process (Supplementary Fig. 4B, C). Despite this interference, DesAb-O did not slow the aggregation process to the same extent as DiDesAb-O, increasing the *t*_50_ value only by 1.3-fold at 1:2 Aβ_42_ monomer:sdAb molar ratio (Supplementary Fig. 4A–C). Notably, DiDesAb-O exhibited a comparable inhibitory effect at a significantly lower molar ratio of 1:0.25 (*t*_50_ value of 1.8 ± 0.5 h), indicating a substantially greater potency of DiDesAb-O in interfering with the aggregation process of Aβ_42_. Again, all *t*_50_ values were determined as the mean of three independent experiments, wherein Supplementary Fig. 4B, C only shows one representative trace per condition.

### DiDesAb-O has enhanced binding sensitivity for Aβ_42_ oligomers relative to DesAb-O

To compare the binding properties of DiDesAb-O and DesAb-O for Aβ_42_ oligomers, we performed a real-time ELISA. To do so, we prepared solutions of 1 μM Aβ_42_, and we monitored the aggregation of Aβ_42_ by ThT fluorescence (Fig. 2B). At different timepoints (0, 0.5, 1, 2, and 22 h), samples were collected and absorbed onto an ELISA plate overnight. The following day, 1 μM DesAb-O or 1 μM DiDesAb-O were used as primary Abs. Immediately after starting the aggregation reaction (0 h), the absorbance was already higher than that found for BSA used as a background signal, probably due to the detection of low molecular weight Aβ_42_ aggregate and, although to a lesser extent, Aβ_42_ monomer by sdAbs (Fig. 2C). After 0.5 h, DiDesAb-O slightly increased the absorbance signal probably due to the increased formation of low molecular weight aggregates, exhibiting a statistically difference with DesAb-O at the same time (Fig. 2C). As the time-course continues, our results revealed a significant increase in absorbance signal for both sdAbs, especially after 1 and 2 h from the beginning of the aggregation reaction, approximately close to the half-time of aggregation as determined by ThT fluorescence (Fig. 2B, C), where oligomers are at their maximum concentration, due to their role as crucial intermediates in fibril formation^46^. At these time-points, DiDesAb-O exhibited higher absorbance than DesAb-O, with absorbance values 1.61 ± 0.2-fold and 1.54 ± 0.30-fold greater than DesAb-O at 1 and 2 h, respectively. Notably, this increase exceeded twice the absolute propagated standard deviation.

In order to understand whether the dimeric form of DesAb-O was recognizing Aβ_42_ oligomers or Aβ_42_ fibrillar conformers, we used Aβ_42_ fibrils obtained after 4 days of incubation at 37 °C as a control, as represented in Fig. 2C. Interestingly, neither sdAbs recognized these Aβ_42_ fibrils, resulting in absorbance signals well below the corresponding values at 0 h and similar to those obtained with BSA. From this evidence, we can assess that DiDesAb-O is able to recognize Aβ_42_ oligomers at very low concentrations, with high specificity and increased sensitivity, representing a successful improvement of the previous DesAb-O binding properties for Aβ_42_ oligomers.

### DiDesAb-O induces morphological changes and increased structural weakness in Aβ_42_ fibrils

**Fig. 3:**
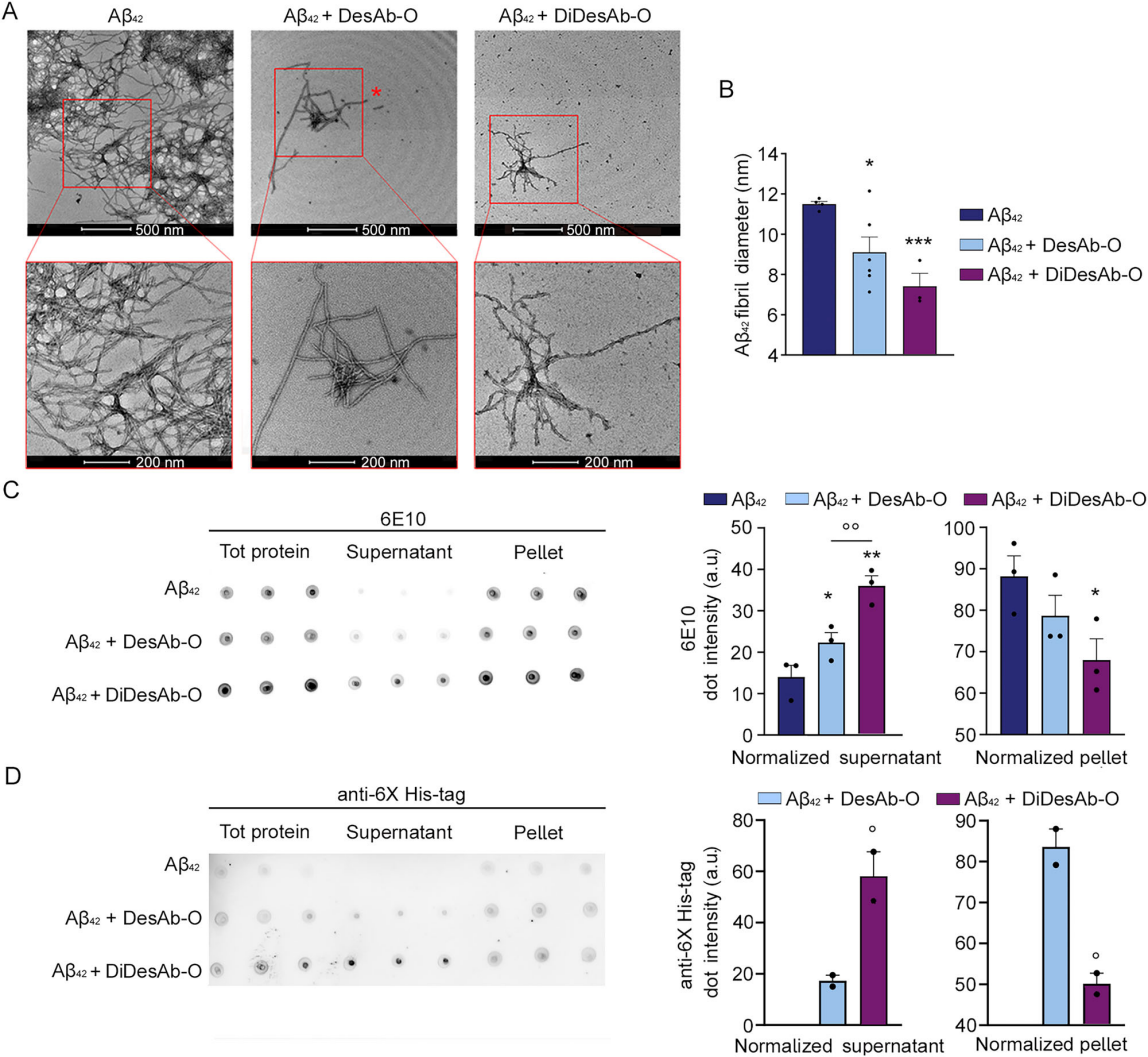
Evaluation of Aβ_42_ fibril fragility and morphological changes induced by co-incubation with sdAbs. **A** TEM image of Aβ_42_ fibrils obtained in the absence or presence of sdAbs after 4 days at 37 °C. Higher magnifications of Aβ_42_ fibrils are shown in the box areas. **B** Aβ_42_ fibrils diameter quantification performed with ImageJ. Error bars are S.E.M. (*n* = 3–6). For each TEM image, 10–30 diameter values were obtained and averaged. Diameter values were compared by one-tailed Student *t* test relative to Aβ_42_ fibrils obtained in the absence of sdAbs (*P < 0.05 and ***P < 0.001, relative to Aβ_42_ fibrils obtained in the absence of sdAbs). **C**, **D** Dot blot of total protein, supernatant and pellet of centrifuged Aβ_42_ fibrils samples obtained with or without co-incubation with sdAbs. The membranes were incubated with 6E10 (**C**) and anti-6X His-tag (**D**) as primary Abs for Aβ_42_ and sdAbs, respectively. Aβ_42_ signal was subtracted as a background. Error bars indicate S.E.M. Statistical analysis was performed by one-tailed Student *t* test. Samples (*n* = 3 for 6E10 Ab and *n* = 2 for anti-6X His-tag Ab, for both supernatant and pellet) were analysed by comparing the absorbance of Aβ_42_ fibrils obtained without Ab VS Aβ_42_ fibrils obtained in the presence of sdAbs (*P < 0.05 VS DesAb-O and **P < 0.01 VS DiDesAb-O, for supernatant and *P < 0.05 VS DiDesAb-O for pellet, respectively) and versus Aβ_42_ fibrils obtained in the presence of DesAb-O compared to Aβ_42_ aggregates obtained in the presence of DiDesAb-O (°°*P* < 0.01). In **D**, the presence of DesAb-O in the supernatant and in the pellet was compared to the presence of DiDesAb-O (°*P* < 0.05 for supernatant and pellet, respectively). Full uncropped and unedited scans of the membranes are provided in Supplementary Fig. 8A.

To further investigate the impact of DiDesAb-O on Aβ_42_ aggregation, we analyzed the morphological changes and fragility alterations induced by the sdAb in Aβ_42_ fibrils. To do so, we aggregated samples containing 5 μM Aβ_42_ in the absence or presence of an equimolar concentration of either DesAb-O or DiDesAb-O for 4 days at 37 °C. Then, we visualized the samples by transmission electron microscopy (TEM) (Fig. 3A). Aβ_42_ aggregated in the absence of sdAbs, forming a dense network of long fibrils with a diameter of 11.5 ± 0.1 nm (Fig. 3A, B). In contrast, Aβ_42_ fibrils obtained in the presence of DesAb-O were shorter and exhibited a reduced diameter of 9.1 ± 0.8 nm (Fig. 3A, B). A striking change in the morphology of Aβ_42_ fibrils was observed when monomeric Aβ_42_ was co-incubated with DiDesAb-O. In this case, the fibrils had a jagged appearance with the presence of apparently globular structures on their surface (Fig. 3A). The fibrils appeared again short and thinner than those formed with DesAbO, with a diameter of 7.4 ± 0.6 nm, suggesting that fibrils may be breaking or degrading due to increased structural weakness. Altogether, these findings suggest a more potent inhibitory effect of DiDesAb-O on fibril assembly and maturation (Fig. 3B).

To further investigate the structural integrity of Aβ_42_ fibrils formed with or without sdAbs, we performed a dot blot assay. Following the end of the ThT fluorescence kinetic experiments, the plate was incubated at 37 °C for 4 days. Samples were then collected and centrifuged to separate the soluble and insoluble protein fractions. Prior to centrifugation, an aliquot of each sample was stored and considered as the total protein amount. To analyze the proportion of soluble and insoluble Aβ_42_ species and Ab fragments, samples were spotted onto a nitrocellulose membrane and incubated with either 6E10 Ab for Aβ_42_ detection or anti-6X His-tag Ab for DesAb-O and DiDesAb-O detection. The signal intensity of the supernatant was normalized to that of the total protein of the corresponding sample. Our results show that Aβ_42_ aggregation in the absence of sdAbs leads to nearly complete conversion of Aβ_42_ monomers into insoluble aggregates, e.g., fibrils, with only 14 ± 3% of protein in the supernatant and 88 ± 5% in the pellet (Fig. 3C). Conversely, samples obtained in the presence of DesAb-O and DiDesAb-O contained an increased amount of Aβ_42_ in the supernatant (22 ± 2% and 36 ± 2%, respectively) and only partial deposition of Aβ_42_ in the pellet (79 ± 5% and 68 ± 5%, respectively), as shown in Fig. 3C. These data indicate that the sdAbs, particularly DiDesAb-O, increased the presence of soluble Aβ_42_ species in the aggregation mixture. This result is in agreement with the presence of small globular aggregates in the background of the TEM images, as noted earlier.

Quantification of the sdAb signals showed that DesAb-O was predominantly in the pellet, likely due to its binding to the aggregates, e.g., the oligomers (17 ± 2% and 84 ± 4% for supernatant and pellet, respectively), whereas DiDesAb-O was largely in the supernatant of the samples (58 ± 9% and 50 ± 3% for supernatant and pellet, respectively) (Fig. 3D). This observation, along with TEM images (Fig. 3A) and ThT fluorescence time-course analyses (Fig. 2A), suggests that DiDesAb-O displays stronger binding to and stabilizes soluble Aβ_42_ aggregates, e.g., the oligomers, which populate the supernatant to the detriment of fibrils. Alternatively, it is also possible that DiDesAb-O can lead to unstable Aβ_42_ fibrils that are more likely to fragment and become soluble.

Finally, we carried out a proteinase K (PK) digestion assay to determine whether the Ab fragments can induce structural modifications in Aβ_42_ fibrils (Supplementary Fig. 5). Fibrils were collected from the co-incubation samples with sdAbs at the end of aggregation after 4 days, treated with increasing concentrations of PK followed by Western blotting analysis was performed using the anti-Aβ_42_ N-terminus 6E10 Ab. Aβ_42_ fibrils formed without sdAbs or with DesAb-O appeared more resistant and did not enter the gel, unlike fibrils formed with DiDesAb-O (Supplementary Fig. 5A). We can assess that all fibrils obtained in different conditions share similar band intensities at 0 μg/mL, demonstrating a consistent baseline across different treatment conditions, enabling reliable normalization of bands obtained at higher PK concentrations (Supplementary Fig. 5B). As far as the fractions that enter the gel, band intensities were normalized to those obtained with 0 μg/mL PK in the corresponding samples, taken as 100%. Aβ_42_ fibrils obtained in the presence of DesAb-O displayed an increased resistance to PK digestion at low PK concentrations (10 μg/mL), probably due to conformational changes in the fibrils, making them slightly more resistant to PK catalyzed cleavage. In contrast, fibrils obtained in the absence of sdAbs and in the presence of DiDesAb-O followed similar PK resistance (Supplementary Fig. 5C).

Overall, these results demonstrate that DesAb-O and DiDesAb-O induce significant changes in the structure of Aβ_42_ fibrils. As evidenced by dot blot, TEM and Western blotting, DiDesAb-O action results in a reduction of fibril diameter and increased structural fragility.

### DiDesAb-O detects Aβ_42_ oligomers interacting with cellular membranes and internalized into the cytoplasm

**Fig. 4:**
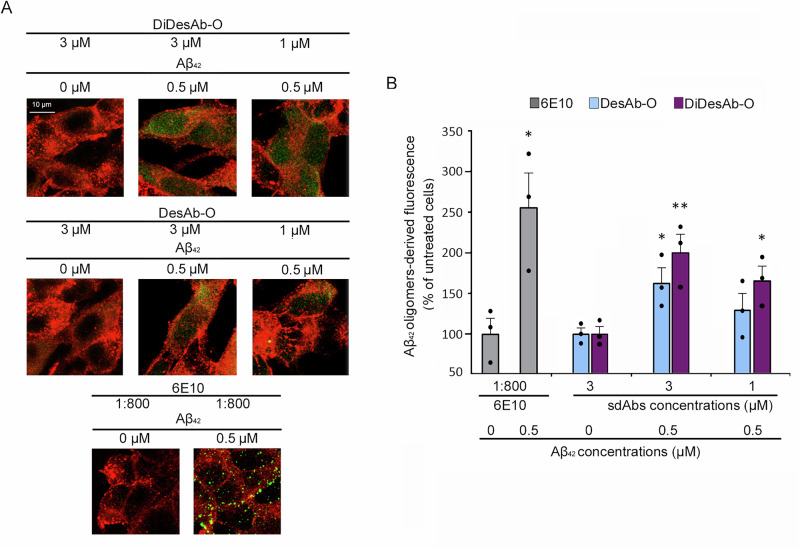
DiDesAb-O detects Aβ_42_ oligomers interacting with neuronal cells and penetrated into the cytosol at lower concentrations than DesAb-O. **A** Representative confocal microscopy image of SH-SY5Y cells left untreated or treated with Aβ_42_ species at 0.5 μM for 1 h. Red and green fluorescence indicates the cell membranes and the Aβ_42_ oligomers, respectively, detected with wheat germ agglutinin (WGA) and sdAbs at two different concentrations (3 or 1 μM), respectively. **B** The bar plot represents the results of a semi-quantitative analysis of green fluorescence. Error bars indicate S.E.M. Statistical analysis was performed by Student *t* test. Sample (*n* = 3) were analysed by Student *t* test to their respective untreated cells (*P < 0.05 and **P < 0.01). 200–250 cells were analyzed per condition.

Next, we aimed to evaluate the ability of DiDesAb-O to detect Aβ_42_ oligomers in cell cultures and inhibit their toxicity using cell biology experiments. For these experiments, we used synthetic Aβ_42_ rather than the recombinant peptide, as it is endotoxin-free, which is a crucial requirement for these sensitive experiments. First, we ascertained the ability of synthetic Aβ_42_ to form toxic aggregates in the absence of sdAbs in a typical time course experiment (10 µM Aβ_42_ in PBS, pH 7.4, 37 °C) (Supplementary Figs. 6 and 7). For the experiments described below with sdAbs, we used the heterogeneous Aβ_42_ aggregate solution formed after 8 h of incubation under the same conditions described above, which we will refer to as “oligomers” as it included mostly oligomeric species that are toxic to the cells. Human neuroblastoma SH-SY5Y cells were exposed to 0.5 μM Aβ_42_ oligomers for 1 h and then treated with sdAbs as primary Abs to detect Aβ_42_ oligomers. Plasma membrane (red channel) and Aβ_42_ oligomers (green channel) were counterstained and analysed by confocal microscopy (Fig. 4A), as previously reported^37^. At 3 μM, both DiDesAb-O and DesAb-O caused a green fluorescence increase in cells treated with oligomers by 200 ± 22% and 163 ± 18%, respectively, as compared to cells treated with sdAbs but not oligomers (Fig. 4B). At 1 μM, only DiDesAb-O was able to produce a significant increase of green fluorescence (166 ± 17% for DiDesAb-O vs. 129 ± 20% for DesAb-O), indicating greater binding to oligomers (Fig. 4B). 6E10 Ab, which was used as a control, can recognize both low and high molecular weight structures, as revealed by the presence of green dots with different size (Fig. 4A). On the contrary, both sdAbs can detect only small oligomeric species within the heterogeneous mixture, representing further evidence of their specificity for Aβ_42_ oligomeric species. Overall, in our experimental conditions, DiDesAb-O selectively recognized toxic Aβ_42_ oligomers interacting with cellular membranes and penetrated into the cytosol even at lower concentrations than DesAb-O, suggesting a stronger binding.

### DiDesAb-O inhibits the interaction of Aβ_42_ oligomers with neuronal membranes

**Fig. 5:**
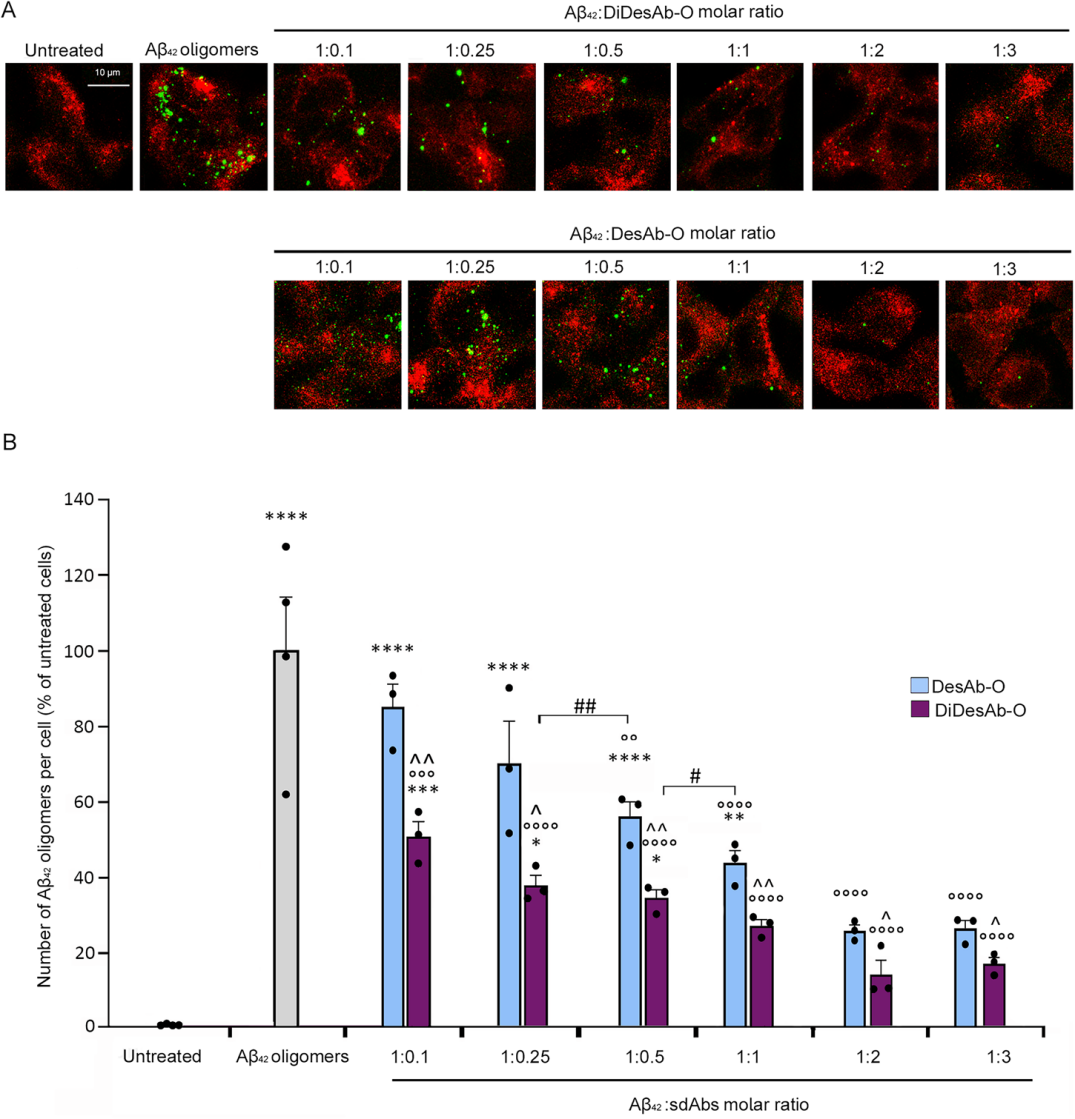
DiDesAb-O inhibits the binding of Aβ_42_ oligomers to the neuronal membrane to a greater extent with respect to DesAb-O. **A** Representative confocal microscopy images of SH-SY5Y cells treated with 0.5 μM Aβ_42_ oligomers following 1 h pre-incubation in the absence (first row) or presence of DiDesAb-O (second row) or DesAb-O (third row) at the indicated Aβ_42_:Abs molar ratios. Red and green fluorescence indicates the cell membranes and Aβ_42_ oligomers detected with WGA and 6E10 Ab, respectively. **B** Counting of Aβ_42_ oligomers bound to the cellular membrane measured following incubation under the conditions represented in (**A**). Error bars indicate S.E.M. Statistical analysis was performed by ANOVA with multiple comparisons or by Student *t* test. Samples (*n* = 3) were analysed by one-way ANOVA followed by Bonferroni’s multiple-comparison test to untreated cells (*P < 0.05, **P < 0.01, ***P < 0.001 and ****P < 0.0001), or to cells treated with Aβ_42_ oligomers only (°°*P* < 0.01, °°°*P* < 0.001, and °°°°*P* < 0.0001). sdAbs at the same molar ratio (^*P* < 0.05 and ^^*P* < 0.01) and at equivalent binding-site concentrations (#*P* < 0.05 and ##*P* < 0.01) were analysed by one-tailed Student *t* test.

We evaluated whether DiDesAb-O was able to neutralize Aβ_42_ oligomers, preventing their interaction with neuronal membranes. To do so, 0.5 μM Aβ_42_ oligomers formed after 8 h of incubation were incubated for 1 h with DesAb-O and DiDesAb-O at varying molar ratios Aβ_42_:sdAbs (1:0.1, 1:0.25, 1:0.5, 1:1, 1:2, and 1:3). These solutions were then added to the cell culture medium of SH-SY5Y cells for 15 min, as previously reported^37^. To detect only the oligomers bound to the cell surface, the cellular membrane was not permeabilized at this stage, thus preventing oligomer internalization. The binding of the aggregates on the cellular membranes was assessed by confocal microscopy using the 6E10 Ab as a probe. Aβ_42_ oligomers colocalized with cellular membranes in the absence of pre-incubation with the sdAbs (Fig. 5A), as previously reported^37,47^. Following the pre-incubation step, the interaction between Aβ_42_ oligomers and the neuronal membranes was significantly reduced (Fig. 5A). In particular, DiDesAb-O appeared to be more efficient than DesAb-O, being more effective at all sdAb concentrations tested and producing the same inhibition as DesAb-O at 10-fold lower concentrations (Fig. 5B). These results suggest DiDesAb-O binds stronger to Aβ_42_ oligomers with respect to DesAb-O.

### DiDesAb-O prevents Aβ_42_ oligomer toxicity

**Fig. 6:**
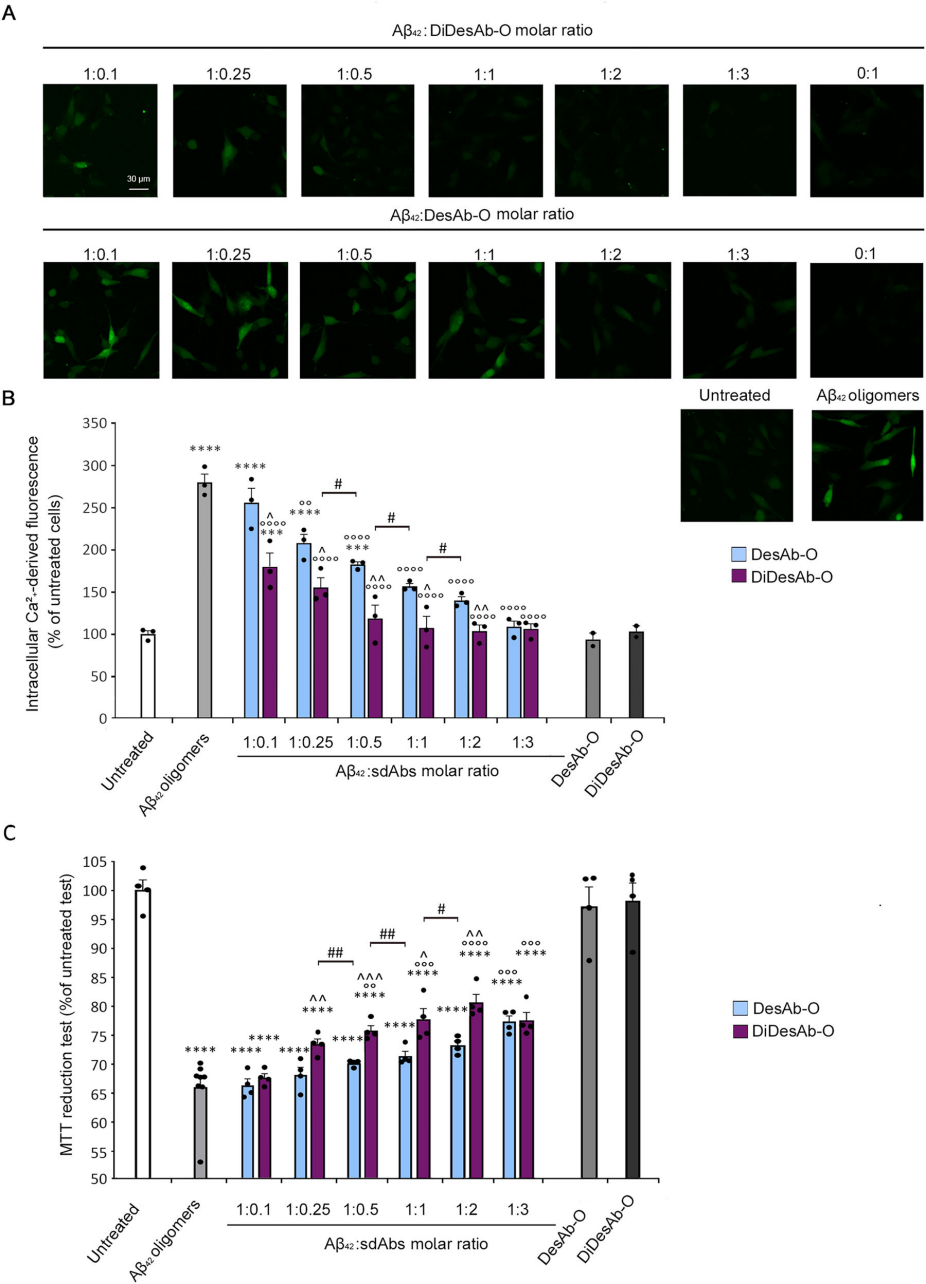
DiDesAb-O strongly prevents the neurotoxicity induced by Aβ_42_ oligomers. **A** Representative confocal microscopy images showing the Ca^2+^-derived fluorescence in SH-SY5Y cells treated for 15 min with 0.5 µM Aβ_42_ oligomers pre-incubated with or without increasing molar ratios (1:0.1, 1:0.25, 1:0.5, 1:1, 1:2, and 1:3) of DiDesAb-O (*top*) or DesAb-O (*bottom*). Cells were then loaded with the Fluo-4AM probe as described in the “Materials and Methods” section. **B** Semi-quantitative analyses of the Ca^2+^-derived fluorescence expressed as the percentage of the value for untreated cells, taken as 100%. **C** MTT reduction in SH-SY5Y cells treated for 24 h with increasing Aβ_42_ oligomers:sdAbs molar ratios (1:0.1, 1:0.25, 1:0.5, 1:1, 1:2, and 1:3). Values are expressed as the percentage of the value for untreated cells, taken as 100%. **B**, **C** Error bars indicate S.E.M. Statistical analysis was performed by ANOVA with multiple comparisons or by Student *t* test. Samples (*n* = 3 for confocal experiment. For MTT assay *n* = 8 for Aβ_42_ oligomers and *n* = 4 for all other conditions, including untreated and antibody only-controls) were analysed by one-way ANOVA followed by Bonferroni’s multiple-comparison test to untreated cells (***P < 0.001 and ****P < 0.0001), or to cells treated with Aβ_42_ oligomers (°°*P* < 0.01, °°°*P* < 0.001 and °°°°*P* < 0.0001). sdAbs at the same molar ratio (^*P* < 0.05, ^^*P* < 0.01 and ^^^*P* < 0.001) and at equivalent binding-site concentrations (#*P* < 0.05 and ##*P* < 0.01) were analysed by one-tailed Student *t* test.

We then evaluated the ability of DiDesAb-O to prevent critical detrimental effects induced by Aβ_42_ oligomers. First, we monitored the disruption of cytosolic Ca^2+^ homeostasis induced by Aβ_42_ oligomers, as previously reported^37,48^. SH-SY5Y cells were treated for 15 min with solutions containing Aβ_42_ oligomers and sdAbs at varying molar ratios. Aβ_42_ oligomers without sdAbs caused an extensive Ca^2+^ influx, with an increase of 280 ± 10% relative to untreated cells (Fig. 6A, B). Pre-incubation of oligomers with DiDesAb-O was found to generate a significant protective effect at 6-fold lower concentration than DesAb-O (Fig. 6A, B). DiDesAb-O was protective even at the lowest concentration tested (0.05 µM, 1:0.1 ratio). Of note, neither sdAb affects neuronal viability when added alone to the cell medium (Fig. 6A, B).

The protective effect of DiDesAb-O was also evaluated by analyzing, with the 3-(4,5-dimethyltohiazol-2-yl)-2,5-diphenyltetrazolium bromide (MTT) reduction test, the mitochondrial status of cultured cells treated with Aβ_42_ oligomers (Fig. 6C). Aβ_42_ oligomeric species at 0.5 μM were incubated in the absence or presence of varying concentrations of sdAbs for 1 h and then added to the culture medium of SH-SY5Y cells for 24 h. While Aβ_42_ oligomers significantly reduced (by 34 ± 2%) the mitochondrial activity of SH-SY5Y cells as compared to untreated cells, as previously reported^37^, pre-incubated with the sdAbs allowed a significant recovery of the mitochondrial functionality (Fig. 6C). Again, DiDesAb-O was more effective than DesAb-O at most Ab concentrations tested. sdAbs alone were, by contrast, ineffective (Fig. 6C).

Considering these results together, we can confirm the increased ability of DiDesAb-O to prevent Aβ_42_ oligomer-induced toxicity.

### DiDesAb-O selectively detects Aβ_42_ oligomers present in the CSF of AD patients and prevents their associated toxic effects

**Fig. 7:**
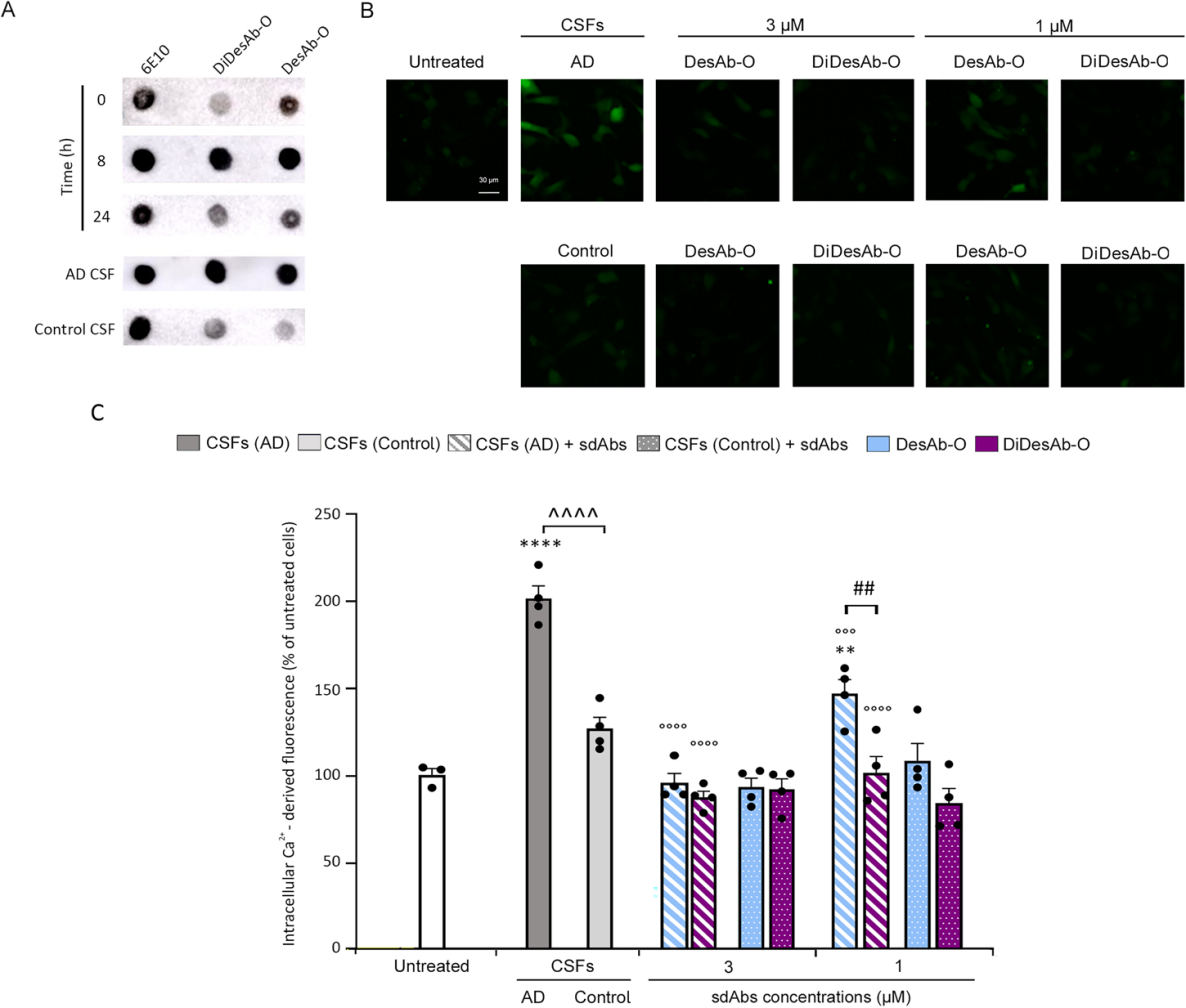
DiDesAb-O significantly reduces the Ca^2+^ dyshomeostasis induced by CSFs derived from AD patients. **A** Representative sandwich dot-blot assay Aβ_42_ species and CSFs. Capture Abs, 6E10 (0.01 mg/mL), DiDesAb-O (10 µM), and DesAb-O (20 µM) were spotted onto nitrocellulose. The membranes were incubated with Aβ_42_ samples collected at 0, 8, and 24 h from a 2 µM aggregation reaction or with CSF from patients and controls at 0.1 mg/mL. Finally, membranes were probed with the detection 6E10 Ab. Full uncropped and unedited scans of the dot blots are provided in Supplementary Fig. 8B**B**, **C** Intracellular Ca^2+-^derived fluorescence in SH-SY5Y cells treated for 5 h with CSFs from AD patients (stripped bars) and age-matched control subjects (dotted bars) (*n* = 4 for AD and *n* = 4 for control subjects), diluted 1:1 with the extracellular medium, following 1 h pre-incubation in the absence (gray bars) or presence of DesAb-O (light blue bars) and DiDesAb-O (purple bars) at 3 or 1 μM, as indicated. Error bars indicate S.E.M. Statistical analysis was performed by ANOVA with multiple comparisons. Samples were analysed by One-way ANOVA followed by Bonferroni’s multiple-comparison test relative to untreated cells (**P < 0.01 and ****P < 0.0001) or to cells treated with AD CSF samples without sdAbs (°°°*P* < 0.001 and °°°°*P* < 0.0001), or to compare AD and controls CSF samples under the same conditions (^^^^*P* < 0.0001) or to compare AD CSF samples with sdAbs tested at the same concentration (##*P* < 0.01).

We assessed whether DiDesAb-O could selectively recognize pathological Aβ_42_ oligomers in CSFs from AD patients compared with healthy age-matched controls using a sandwich dot-blot previously developed^37^. Briefly, 2 µL of the capture antibodies 6E10 (0.01 mg/mL), DiDesAb-O (10 µM), and DesAb-O (20 µM) were spotted onto nitrocellulose membranes. The membranes were then incubated either with Aβ_42_ samples collected at 0, 8, and 24 h from a 2 µM aggregation reaction (Supplementary Fig. 6) or with CSFs from patients and controls at 0.1 mg/mL. The antibody 6E10 was used as a control for equal loading, as it binds Aβ_42_ independently of its aggregation state. DiDesAb-O gave its strongest signal with the 8 h Aβ_42_ sample, a minor signal with the 0 h sample, and only a weak signal with the 24 h sample. DesAb-O also reacted most strongly with the 8 h sample, but displayed a clearer signal at 0 h, consistent with partial monomer binding^36,37^. Importantly, DiDesAb-O produced an intense signal with AD CSFs and only a weak signal with control CSFs (Fig. 7A), supporting its potential to discriminate between AD and age-matched control subjects.

We then performed a proof-of-concept experiment on a small set of clinical samples of CSF (*n* = 4 from AD patients and *n* = 4 from controls subjects) to evaluate whether DiDesAb-O was also able to neutralize the cytotoxicity induced by Aβ_42_ oligomeric species present in AD CSFs and whether the engineering of the DiDesAb-O sdAb into a dimeric form resulted in improved performance with respect to the monomeric sdAb (Fig. 7B, C). Thus, we monitored the dysregulation of cytosolic Ca^2+^ homeostasis in SH-SY5Y treated for 5 h with the CSFs from AD patients and control subjects diluted 1:1 with the cell culture medium, in the absence or presence of a 1 h pre-incubation with 3 or 1 μM sdAbs, as previously reported for the monomeric sdAb^37^. The AD CSFs significantly increased the intracellular Ca^2+^ concentration by 201 ± 7% with respect to untreated cells, whereas control CSFs induced a nonsignificant increase to 127 ± 6% (Fig. 7B,C). After 1 h of pre-incubation with 3 μM DesAb-O and 3 μM DiDesAb-O, both sdAbs completely reduced the Ca^2+^ dyshomeostasis induced by AD CSFs (96 ± 5% and 87 ± 3% relative to untreated cells, respectively) (Fig. 7C). After 1 h of pre-incubation with 1 μM DesAb-O, the sdAb lost in part its efficacy, whereas DiDesAb-O continued to be active against AD CSFs (Fig. 7C).

Taken together, these data demonstrate the ability of DiDesAb-O to selectively detect and neutralize toxic species present in the CSFs of AD patients, to an extent larger than that of the monomeric counterpart.

## Discussion

Protein aggregates, characterized by extreme structural heterogeneity, play a central role in many neurodegenerative diseases, yet the development of molecular probes and therapeutics capable of selectively targeting these species remains a major challenge. Over the past two decades, several mAbs have been developed to detect or remove Aβ aggregates in the brains of AD patients^20^. Some of these, such as lecanemab and donanemab, have received FDA approval^25,27^. Despite their clinical potential, many trials have failed, and even the FDA-approved mAbs are controversial due to their severe side effects^25–27^. The large molecular size of conventional mAbs (~150 kDa) significantly limits their ability to cross the BBB, as only a small fraction (typically less than 0.1%) reaches the CNS. This low bioavailability reduces their efficacy in targeting Aβ aggregates in the brain. Furthermore, mAbs have a significant immunogenicity and off-target binding, potentially leading to side effects.

Ab fragments, such as sdAbs, represent highly promising biomolecules for diagnostic and therapeutic applications for a range of neurological disorders, including dementia. They offer several advantages over mAbs, including high solubility, low immunogenicity^28–31^. Furthermore, sdAbs can be engineered with a variety of protein engineering approaches to enhance different properties, such as the BBB penetration^49–51^ and microglia activation for degradation of neurotoxic aggregates from brain parenchyma^38^. To date, numerous Ab fragments have been developed against various types of Aβ aggregates, showing promising results in preclinical studies by neutralizing Aβ aggregate toxicity^28,52–55^. Beyond therapeutic applications, sdAbs, such as R3VQ and A2, are effective in vivo imaging agents due to their ability to bind Aβ and tau aggregates^56^. While all these molecules are promising, they often recognize nontoxic monomers and/or less-toxic amyloid fibrils^36^. Thus, higher specificity for the oligomers is required to enable clinical applications. Additionally, most of these molecules are derived from nonhuman framework sequences, which may trigger the formation of anti-drug Abs (ADA) when administered to humans regularly^57^. Thus, Ab fragments consisting of human-derived Ab domains may find more streamlined clinical applications.

Building on the promising functional properties of the previously characterized sdAb, DesAb-O^36,37^, here we present a strategy to enhance its avidity and binding ability for Aβ_42_ oligomers by designing an improved dimeric version, named DiDesAb-O. Our hypothesis was that Aβ_42_ oligomers present multiple copies of the same target epitope. By making DesAb-O dimeric, it could simultaneously bind to multiple epitopes, thereby increasing binding strength through avidity. The use of flexible linkers to engineer sdAbs for enhanced binding avidity and specificity has been successfully demonstrated^58,59^. In our approach, we created a dimeric structure by linking two monomers with a (GGGGS)₃ linker.

Our functional analyses revealed that DiDesAb-O displays stronger oligomer binding and anti-aggregation activity compared to monomeric DesAb-O. Furthermore, it can induce drastic morphological changes in Aβ_42_ fibrils. Other Ab fragments specific for Aβ_42_ oligomers, such as V31-1^28^, A4-scFv, and E1^53^, have been shown to inhibit fibril formation. Specifically, dynamic light scattering (DLS) showed an inhibitory effect of V31-1^28^, whereas AFM analysis confirmed the same property for A4-scFv and E1^53^.

We also showed the ability of DiDesAb-O to selectively detect Aβ_42_ oligomers in cultured cells, following the approach of our previous work with DesAb-O^37^. We found that pre-incubation of Aβ_42_ oligomers with DiDesAb-O resulted in a significant reduction of their interaction with neuronal membranes, more effectively than with DesAb-O. Furthermore, we observed reduced oligomer-induced intracellular Ca^2+^ flux and improved prevention of the mitochondrial dysfunction compared to DesAb-O, suggesting an enhanced detection of key epitopes in the structure of the toxic oligomers.

Prior to this work, other mAbs^60,61^ or Ab fragments^54,62,63^ demonstrated their ability to counteract Aβ_42_ oligomer-induced toxicity. However, from all these studies, it is difficult to extrapolate the exact type of aggregate species in the milieu of metastable species recognized by Abs or Ab fragments. At present, only V31-1^28^, A4-scFv, E1^53^, DesAb-O^36^ and DiDesAb-O studied here, target specifically Aβ_42_ oligomers. Given that targeting specific conformational Aβ species can lead to markedly different outcomes, our study focuses on enhancing the binding ability of DesAb-O to more effectively target toxic oligomers. Improving oligomer specificity may not only increase therapeutic efficacy but also allow for lower dosage requirements, potentially minimizing the risk of adverse effects.

To evaluate the improved specificity in a biologically relevant context, we conducted further analyses to determine whether DiDesAb-O could specifically bind to neurotoxic Aβ_42_ oligomers present in AD CSFs and effectively neutralize their toxic effects on neuroblastoma cells. DiDesAb-O was shown to prevent neuronal dysfunction caused by Aβ_42_ oligomers in the CSFs of AD patients at lower concentrations than DesAb-O. This finding highlights its enhanced sensitivity for Aβ_42_ oligomers and its capability to detect these toxic species within complex biological fluids, such as AD CSF.

Overall, this study demonstrates that the engineering of sdAbs and, in particular, the rational design of dimeric sdAbs, can significantly enhance their binding toward defined pathological protein species, e.g., Aβ_42_ oligomers. Although further validation in larger and independent patient cohorts will be required, these findings provide a robust foundation for the development of next-generation Ab fragments with enhanced targeting to heterogeneous protein aggregates. This approach holds significant promise for enabling earlier diagnosis, timely therapeutic intervention, and improved disease management in AD and other protein misfolding-related neurodegenerative diseases.

## Method

### DiDesAb-O design

To design DiDesAb-O, a flexible linker composed by a repetition of glycine (Gly) and serine (Ser), was attached to the C-terminus of a first DesAb-O monomer and the N-terminus of a second DesAb-O monomer, depleted of the initial four amino acids (methionine (Met), arginine (Arg), Gly, and Ser), and the following 6X His-tag region. The precise sequence of the linker was GGGGSGGGGSGGGGS. Gene sequence design was carried out through the use of SnapGene software (www.snapgene.com).

### Expression and purification of DesAb-O and DiDesAb-O

DesAb-O was expressed using a pRSET-B vector^36^ in *E. coli* Origami 2 (DE3) cells (Merck Millipore). Cells were grown at 37 °C in Luria–Bertani (LB) medium (Merck Millipore) supplemented with ampicillin (100 μg/mL) under shaking at 200 rpm in a New Brunswick Innova 44R incubator shaker (Eppendorf) until reaching an OD_600_ of 0.6. Then, protein expression was induced with 1 mM IPTG overnight at 18 °C with shaking at 200 rpm. DiDesAb-O was expressed using a pET28a (+) vector in *E. coli* Origami 2 (DE3) cells (Merck Millipore). Cells were grown under the same conditions except that the medium was supplemented with Kanamycin (50 μg /ml). Cells were then harvested by centrifugation, resuspended with 20 mM phosphate buffer, pH 8.0, with the addition of one EDTA-Free Complete Protease Inhibitor Cocktail Tablet (Roche) per 500 ml of culture, and lysed using sonication (15 s on and 45 s off pulses, 40% amplitude). Cell debris was removed using centrifugation at 20,000 × *g* (JA-20 rotor, Beckman Coulter) for 45 min. The cleared supernatant lysate was loaded onto a Ni^2+^-NTA HisTrap Superflow column (Cytiva), previously equilibrated with 20 mM phosphate buffer, pH 8.0, containing 15 mM imidazole. After washing with 20 mM phosphate buffer containing 30 mM imidazole, the His-tagged DesAb-O and DiDesAb-O were eluted with 20 mM phosphate buffer containing 300 mM imidazole and dialyzed extensively against 20 mM phosphate buffer. DesAb-O and DiDesAb-O were finally purified using size-exclusion chromatography with either a HiLoad^TM^ 26/600 Superdex 75 pg column (Supplementary Fig. 1) or a HiLoad^TM^ 16/600 Superdex 75 pg column (both from Cytiva), previously equilibrated in 20 mM phosphate buffer, pH 8.0. Protein concentration was determined by absorbance measurement at 280 nm using theoretical extinction coefficients calculated with ExPASy ProtParam. Both the flow through and peak fractions were then loaded on 4–12% Bis-Tris NuPAGE gels (Thermo Fisher Scientific) to verify the sample purity.

### Expression and purification of Aβ_42_

The Aβ_42_ peptide fused to the spider silk domain (known as fusion protein, 20 kDa) was expressed in BL21 *E. coli*^64,65^. Cells were grown at 37 °C in LB broth supplemented with kanamycin (50 μg/mL) with shaking at 200 rpm in a New Brunswick Innova 44R incubator shaker (Eppendorf) until an OD_600_ of 0.8 was reached. Protein expression was then induced with 1 mM IPTG, and cultures were incubated overnight at 20 °C with shaking at 200 rpm. Then, cells were collected, centrifuged, and resuspended in 20 mM Tris−HCl and 8 M urea, pH 8.0 to be sonicated on ice for 20 min (15 s on and 45 s off pulses, 20% amplitude). Following another centrifugation, the supernatant was passed through a 0. 22 μm filter and loaded onto two tandem 5 ml HisTrap HP columns (Cytvia), pre-equilibrated with a binding buffer (20 mM Tris-HCl, 8 M urea, pH 8 supplemented with 15 mM imidazole)^65^. After washing the columns with the same buffer to remove nonspecific binding, the fusion protein was eluted using an elution buffer 20 mM Tris-HCl, 8 M urea, pH 8.0 supplemented with 300 mM imidazole (elution buffer)^65^. The elute underwent overnight dialysis against 20 mM Tris−HCl (pH 8.0), followed by concentration measurements via Nanodrop. To release the target peptide, the fusion protein was incubated with TEV protease (1:15 molar ratio) overnight at 4 °C. Subsequently, the sample was treated with 7 M guanidine-HCl on ice for at least 2 h. Purification of the monomeric Aβ_42_ was performed by applying the sample onto a Superdex^TM^ 75 Increase 10/300 GL column (Cytiva) pre-equilibrated with 20 mM phosphate buffer supplemented with 200 μM EDTA, pH 8.0, for size-exclusion chromatography^65^. The monomeric peak was collected manually, and its concentration was calculated based on the absorbance at 280 nm using the following equation^65^:

1 $${Concentration}\,\left({{{\mathrm{\mu M}}}}\right)=\left(\frac{{A}_{280}/2}{0.2\,\times 1490\,}\right)\times {10}^{6}$$

Where:

- 0.2 cm represents the path length of the AKTA pure system
- 1490 M^−1^ cm^−1^ is the molar extinction coefficient of Aβ_42_.

Finally, the stock was adjusted to the target concentration required for all subsequent experimental assays. For all cellular biology experiments, we purchased synthetic Aβ_42_ peptides from Bachem to obtain transient oligomers from in vitro aggregation assay and ADDLs oligomers^43^.

### Preparation of ADDLs

The lyophilized synthetic Aβ_42_ peptide (Bachem) was dissolved in 100% hexafluoro-2-isopropanol (HFIP) to obtain a monomeric form, followed by evaporation under nitrogen flux. To obtain ADDLs, Aβ_42_ was resuspended in anhydrous dimethyl sulfoxide (DMSO) to 5 mM and then diluting in ice-cold F-12 medium to a final concentration of 100 μM. This solution was incubated at 4 °C for 1 day and then centrifuged at 14,000 × *g* for 10 min^43^. The resulting supernatant, containing the soluble ADDLs, was collected and used for further experiments^43^.

### Structural characterization by CD

Far-ultraviolet (far-UV) CD spectra of DiDesAb-O were acquired using a Chirascan spectropolarimeter (Applied Photophysics) equipped with a Peltier temperature control unit. Measurements were performed in a quartz cuvette with 1 mm path length. Samples contained 6 μM protein in 10 mM Na_2_HPO_4_, 1.8 mM KH_2_PO_4_, 137 mM NaCl and 2.7 mM KCl, pH 7.4 (phosphate-buffered saline or PBS). The far-UV CD spectra of DiDesAb-O were recorded from 200 to 240 nm at 20 °C, and the spectrum of the buffer was subtracted from the spectra of DiDesAb-O. Accumulation was 10. In another set of experiments, CD spectra of 6 μM DesAb-O or DiDesAb-O were recorded between 20 and 90 °C with 5 °C intervals. A background spectrum of the sample buffer was subtracted from all sample spectra. Raw data of *θ* (units of mdeg) were converted to mean residue ellipticity ([*θ*]_res_, units deg cm^2^ dmol^−1^) using^66^:

2 $${[\theta ]}_{{{\mathrm{res}}}}=\theta /[(n-1)\cdot l\cdot c]$$

where [*θ*]_res_, is in deg cm^2^ dmol^−1^, *θ* is in mdeg, *n* is the number of amino acid residues, *l* is the cuvette pathlength in mm, and *c* is the protein concentration in M. The denaturation curves were obtained by plotting [*θ*]_res_ against temperature, fitted with the Santoro and Bolen^67^ equation and normalized to fraction folded (%) values:

3 $$[\theta ]{res}=\,\frac{[\left([\theta ]{res}\left(F\right)+m\left(F\right)*T\right)+\left([\theta ]{res}\left(U\right)+m\left(U\right)*T\right)*{e}^{\left(\frac{-\,\Delta G}{{RT}}\right)}]}{[1+\,{e}^{\left(\frac{-\,\Delta G}{{RT}}\right)}]}$$

where [*θ*]_res_ is the measured molar residue ellipticity at temperature *T* (°C), [*θ*]_res_(*F*) and [*θ*]_res_(*U*) are the [*θ*]_res_ values for the folded and unfolded states at 20 °C, respectively, *m(F)* and *m(U)* are the slopes of the folded and unfolded baselines, respectively, Δ*G* is the Gibbs free energy change upon unfolding and *R* is the universal gas constant.

By fitting the data obtained with the above equation, it was possible to determine the Δ*G* with the temperature increase. In order to calculate the temperature of half-denaturation (*T*_*m*_), we firstly determined the fraction folded with the following equation:

4 $${Fraction}\,{folded}\,\left( \% \right)=\frac{\left(\left[\theta \right]{res}\,-\,\left[\theta \right]{res}\left(U\,\right)\right)}{\left(\left[\theta \right]{res}\left(F\,\right)-\,\left[\theta \right]{res}\left(U\,\right)\right)}$$

The Eq. (3) works by normalizing the observed signal [*θ*]_res_ against the known spectral endpoints. The denominator ([*θ*]_res_(*F*)-[*θ*]_res_(*U*)) establishes the maximum possible signal change between the fully folded ([*θ*]_res_(*F*)) and fully unfolded ([*θ*]_res_(*U*)) states. The numerator measures how far the observed signal is from the completely unfolded state, allowing the calculation of the fraction that has successfully attained the native conformation. The *T*_*m*_ values represent the temperature at which the protein is 50% folded and 50% unfolded (fraction folded = 50%).

### SPR analysis

SPR measurements were performed on a Biacore X100 instrument (Cytiva). The ADDLs (ligand) was immobilized on a carboxymethyl dextran (CM5) sensor chip via amine coupling^44,45^. The CM5 chip was first activated with 100 mM N-hydroxysuccinimide (NHS) and 500 mM 1-ethyl-3-(3-dimethylaminopropyl)carbodiimide (EDC) using a contact time of 420 s and a flow rate of 10 µL min^−1^. Subsequently, the CM5 surface was modified with ADDLs (10 µM in 10 mM acetate buffer, pH 4.0, contact time of 1080 s), followed by deactivation with 1 M ethanolamine (EA, contact time of 420 s). The sensor chip was then equilibrated with PBS (pH 7.4) at a constant flow rate of 5 µL min^−1^ and 37.0 ± 0.5 °C.

Single-cycle (SCK) analysis was performed for both DiDesAb-O and DesAb-O (analytes). SCK consisted of sequential injections (180 s each) of five increasing analyte concentrations (0.625–10.0 nM for both sdABs, and also 58–930 nM for the DesAb-O) over the chip surface. The full binding curves were recorded in real time; however, responses at a defined stability time point were subsequently used to calculate the apparent dissociation constant (*K*_*D*_*)* (Supplementary Table 2). Each measurement was performed in triplicate, and raw sensograms are reported in Supplementary Fig. 3. After each of the three measurement cycles, the ADDLs surface was regenerated by two short pulses (30 s each) of 10 mM HCl. The instrument was operated with two channels in series for simultaneous measurements. Double referencing was applied to correct for bulk effects and other systematic artifacts (subtraction of the reference surface and blank injections). The apparent *K*_*D*_ for DiDesAb-O was calculated from the average binding responses at the defined stability time point using the one-site binding model^45^:

5 $${RU}={RU}_{\max }\times \frac{[{Analyte}]}{\left[{Analyte}\right]+{K}_{D}}$$

where RU is the SPR response and RU_max_ is the maximum SPR response, [analyte] is the DiDesAb-O concentration, and *K*_*D*_ corresponds to the analyte concentration yielding half of the maximum response (RU_max_/2). The *K*_*D*_ for DesAb-O was calculated using the same approach, except by fixing the RU_max_ value at 5.5, which is that determined for DiDesAb-O, allowing a proper comparison. Data analysis and graphing were performed using OriginPro (version 2025, OriginLab Corporation, Northampton, MA, USA).

### ESI-MS

Purified DiDesAb-O (∼20 µM) was analysed by ESI-MS to confirm molecular weight and sample purity. ESI-MS was performed by the Chemistry Mass Spectrometry facility at the Molecular Sciences Research Hub, Imperial College London.

### ThT fluorescence assays

Monomeric recombinant Aβ_42_ (1 μM) was incubated in the absence or presence of DiDesAb-O at decreasing molar ratios (1:1, 1:0.5, 1:0.25, 1:0.125, corresponding to 1, 0.5, 0.25, 0.125 μM DiDesAb-O concentrations) in 20 mM phosphate buffer, pH 8.0, 37 °C. Samples were prepared with a final concentration of 10 μM ThT dye, gently vortexed, and pipetted into nonbinding surface black 96-well plates (Greiner Bio-One) in triplicates. The plate was read in a ClarioStar Plus microplate reader (BMG LabTech) at 37 °C. The excitation and emission wavelengths were set to 440 and 480 nm, respectively, and fluorescence intensity measurements were taken using spiral averaging (3 mm diameter). Buffer-only values were not subtracted from the sample readings but shown in the raw data analysis. Readings were taken every 2 min. To test the capability of DiDesAb-O compared to DesAb-O, in another set of experiments monomeric Aβ_42_ (1 μM) was incubated alone or in the presence of 1 μM DiDesAb-O or increasing DesAb-O molar ratios (1:1, 1:2 corresponding to 1 and 2 μM DesAb-O concentrations) under the same conditions.

To perform in vitro aggregation assays for cell biological experiments, the lyophilized synthetic peptide (Bachem) was resuspended in PBS, pH 7.4, resulting in a final concentration of 10 µM. For visualization of the emerging β-sheets in Aβ_42_, samples were added with a final concentration of 25 µM ThT, gently vortexed and pipette into nonbinding surface black 96-well plates (Grenier Bio-One) in quadruplets. The plate was read in a BioTek Synergy^TM^ H1 Hybrid Multi-mode reader (Agilent) at 37 °C. The excitation and emission wavelengths were set to 440 and 485 nm, respectively. Buffer-only values were not subtracted from the sample readings but shown in the final graph. Readings were taken every 2 min. All data were plotted using GraphPad Prism version 9.3.1 for Windows (GraphPad Software). To characterize the different types of aggregates formed during the Aβ_42_ aggregation process, we collected Aβ_42_ samples at various timepoints (0, 2, 4, 8, and 24 h) to conduct further experiments (see details below on the subsections of Supplementary Figs. 6 and 7).

### Real-time ELISA

Real-time ELISA experiments were performed aggregating 1 μM monomeric recombinant Aβ_42_ peptides in 20 mM sodium phosphate buffer, pH 8.0 in quiescent conditions, at 37 °C. Aliquots of 20 µl were taken at precise timepoints (0, 0.5, 1, 2, and 20 h) from aggregation reactions and immobilized on a 96-well Maxisorp ELISA plate (Nunc) with no shaking overnight at 4 °C. Aβ_42_ fibrils obtained after 4 days of incubation at 37 °C were used as a control. At the end of the incubation, the plate was then washed three times with TBS (20 mM Tris, pH 7.4, and 100 mM NaCl) and incubated in TBS supplemented with 5% bovine serum albumin (BSA) under constant shaking for 1 h at RT. The plate was then washed six times with TBS and then incubated with 30 μL solutions 1 μM DesAb-O or 1 μM DiDesAb-O under constant shaking either for 1 h at RT or overnight at 4 °C. At the end of this incubation, additional six washes with TBS were performed, and the plate was incubated with 30 μL solutions of rabbit polyclonal 6X His tag horseradish peroxidase (HRP) conjugated (Abcam) at a dilution of 1:4.000 in 20 mM Tris, pH 7.4, 100 mM NaCl, and 5% BSA under constant shaking for 1 h at RT. Finally, the plate was washed twice with 20 mM Tris, pH 7.4, and 100 mM NaCl, then three times with 20 mM Tris, pH 7.4, 100 mM NaCl, and 0.02% Tween-20 and again three times with 20 mM Tris, pH 7.4, and 100 mM NaCl. Finally, the amount of bound sdAbs was quantified by using 1-step Ultra TMB-ELISA substrate solution (Thermo Fisher Scientific), according to the manufacturer’s instructions, and the absorbance was measured at 450 nm by means of a CLARIOstar plate reader (BMG Labtech)^36^. Fold change (FC) has been calculated as the ratio of the absorbance values (DiDesAb-O/DesAb-O) at each time-point. The propagated standard deviation (*σ*_FC_) was obtained using the following equation:

6 $${\sigma }_{{{{\boldsymbol{FC}}}}}={{{\boldsymbol{FC}}}}\,\times \root{{{{{\bf{2}}}}}}\of{{\left(\frac{{{{\boldsymbol{\sigma }}}}{{{\boldsymbol{A}}}}}{{{{\boldsymbol{A}}}}}\right)}^{{{{\bf{2}}}}}+{\left(\frac{{{{\boldsymbol{\sigma }}}}{{{\boldsymbol{B}}}}}{{{{\boldsymbol{B}}}}}\right)}^{{{{\bf{2}}}}}\,}$$

Where:

- *A* and *B* correspond to the averaged absorbance signals for DesAb-O and DiDesAb-O, respectively.
- *σA* e *σB* represent the standard deviation of DesAb-O and DiDesAb-O, respectively.

### TEM imaging

Aβ_42_ recombinant samples incubated at 5 μM in the absence or in the presence of equimolar concentration of sdAbs were induced to aggregate in 20 mM sodium phosphate buffer, pH 8.0, in a microplate without ThT and collected after 24 h of aggregation at 37 °C. Aβ_42_ aggregation was monitored by other replicates with ThT, allowing real-time monitoring of the reaction. Samples for TEM were then prepared spotting 4 μL onto Formvar/carbon-coated 300 mesh copper grids for 1 min. By blot drying the grid with Whatman filter, we removed excess sample, allowing the grid to dry for 2 min. Samples were then washed with 4 μL of water and stained with 4 μL of 2% w/v uranyl acetate^65^, and the grid was dried again as described above. Grids were imaged on a T12 Spirit electron microscope (Thermo Fisher Scientific). The diameters of various fibrils were measured using ImageJ software, and all data were plotted using Excel (Version 16.89.1 (24091630)).

### Sandwich dot blot analysis

Sandwich dot-blot was performed by spotting 2 µl of 6E10, DiDesAb-O and DesAb-O Abs (0.01 mg/ml, 10 and 20 µM, respectively) onto nitrocellulose membranes^37^. After 20 min, the blots were blocked in TBS-Tween-20 0.2% and 2.5% BSA IgG free for 40 min. The membranes were incubated with different Aβ_42_ species (2 µM) obtained at different timepoints (0, 8, and 24 h) from the ThT aggregation assay, or with the CSF samples (CSFs) of AD patients and control subjects (see below for details) at 0.1 mg/ml. Then, the membranes were probed with 1:1000 diluted 6E10 Ab overnight at 4 °C under constant shaking. The following day, the membranes were washed three times in TBS-Tween-20 0.2% and incubated with 1:3000 diluted rabbit anti-mouse IgGHRP-conjugated to HRP for 1 h. After three additional washes, the immunolabelled dots were detected using a SuperSignal West Dura (Pierce) ImageQuant™ TL analysis software (GE Healthcare UK Limited version 8.2)^37^.

### Dot blot analysis

To investigate the structural integrity of Aβ_42_ fibrils formed with or without sdAbs, a dot blot analysis was conducted. Following the end of the ThT fluorescence kinetic experiments, the plate was incubated at 37 °C for 4 days. Samples were then collected and centrifuged at max speed (∼17,000 × *g*) for 30 min on a benchtop centrifuge to separate the soluble and insoluble fractions^65^. Prior to centrifugation, an aliquot of each sample was stored and considered as the total protein amount. To analyze the proportion of soluble and insoluble Aβ_42_ species and Ab fragments, three repeats of each samples were spotted onto a nitrocellulose membrane and blocked in 5% nonfat milk in 0.1% PBS-Tween for 45 min at RT^65^. The membranes were then incubated overnight at 4 °C with either 1:1000 6E10 Ab for Aβ_42_ detection or 1:1000 anti-6X His-tag Ab for DesAb-O and DiDesAb-O detection. The following day, the membrane were washed three times for 10 min each in 0.1% PBS-Tween. Membranes were then incubated for 1 h at RT protected from light with 1:2000 anti-mouse Alexa fluor 647 (Thermo Fisher Scientific) for 6E10 Ab and 1:1000 anti-goat anti-6X His Tag (Abcam) for the anti-6X His Tag Ab, respectively. After three additional 10-minute washes in 0.1% PBS-Tween, the membranes were imaged with a Typhoon scanner (GE Healthcare) using the appropriate laser settings^65^. The signal intensity of the supernatant was normalized to that of the total protein of the corresponding sample.

In another set of experiment to characterize the different types of Aβ_42_ aggregates formed during the aggregation process of the synthetic peptide (Bachem), 2.0 µl (equivalent to 0.1 µg) of each sample were collected at five different timepoints (0, 2, 4, 8, and 24 h) and spotted onto a nitrocellulose membrane. Following a 45-min blocking step (1.0% bovine serum albumin, BSA, in TBS-Tween 0.1%), the membrane was incubated with 1:15.000 diluted human monoclonal anti-ADDLs (19.3) Ab (Creative Biolabs), 1:1000 diluted rabbit polyclonal anti-amyloid fibrils (OC) Ab (Sigma-Aldrich) and 1:800 6E10 Ab for 1 h and 30 min. Subsequently, the membrane was washed three times in TBS-Tween 0.1% for 10 min each and incubated with 1:3000 diluted goat anti-6X His tag (Abcam), goat anti-human (Sigma-Aldrich), or goat anti-rabbit (Abcam) or rabbit anti-mouse (Abcam) Abs, all conjugated with horseradish peroxidase (HRP) for 1 h. After three additional washes in TBS-Tween 0.1%, the immunolabelled dots were detected using a Super-Signal West Dura (Pierce) ImageQuant™ TL software (GE Healthcare UK Limited version 8.2)^37^.

### PK digestion and Western blotting

Fibrils of recombinant Aβ_42_ (5 µM) obtained after 4 days at 37 °C under constant conditions in the absence or in the presence of sdAbs were centrifuged at max speed (∼16,000 × *g*) for 1 h, and the supernatant was discarded. The pellet was resuspended in 20 mM phosphate buffer and treated with increasing PK concentrations (0, 10, 25, and 50 µg/ml) for 30 min at RT. Samples were then incubated at 95 °C for 5 min to stop the enzymatic reaction, loaded on 4–12% Bis-Tris NuPAGE gels and transferred onto a 0.45 μm nitrocellulose membrane for 7 min at 20 V with the iBlot 2 (Thermo Fisher Scientific). Membranes were blocked with 5% non-fat dry milk in 0.1% PBS-Tween for 45 min at room temperature^65^, followed by overnight incubation at 4 °C with the 6E10 Ab (1:1000 dilution in 0.1% PBS-Tween). The following day, membranes was washed three times in TBS-Tween 0,1% for 10 min each and incubated for 1 h at RT with 1:2000 anti-mouse Alexa fluor 647 (Thermo Fisher Scientific). After three additional washes, the detection were carried out with a Typhoon scanner (GE Healthcare) using the appropriate laser settings^65^. Data analysis was performed setting the band intensity at 0 µg/ml PK of each sample as the 100%. Gross values of band intensities of 0 µg/ml PK samples were compared as well.

### CSF samples

CSFs from human aged controls (n = 4) or AD patients (n = 4) were commercially obtained from BioIVT (New York, USA). All samples were anonymised prior to receipt, and no identifiable personal data were accessible to the investigators. The supplier confirmed that specimens were collected under informed consent and appropriate ethical approval at the point of collection. Each CSF was received in 0.5–1 ml aliquot and stored at −80 °C. Samples were centrifuged at 4000 × *g* for 10 min at 4 °C, obtaining a pale pellet that was separated from the supernatant^37^. The supernatant was then analysed; protein concentration in these samples was determined by the Bradford colorimetric method^68^.

### Cell cultures

Authenticated human SH-SY5Y neuroblastoma cells were purchased from A.T.C.C. and cultured in Dulbecco’s modified Eagle’s medium (DMEM), F-12 Ham with 25 mM 4-(2-hydroxyethyl) piperazine-1-ethanesulfonic acid (HEPES) and NaHCO_3_ (1:1) supplemented with 10% fetal bovine serum (FBS), 1.0 mM glutamine and 1.0% penicillin and streptomycin solution (Sigma-Aldrich)^69,70^. Cells were maintained in a 5.0% CO_2_ humidified atmosphere at 37 °C and grown until 80% confluence for a maximum of 20 passages and tested to ensure that they were free from mycoplasma contamination^37,70^.

### Confocal microscopy

Synthetic Aβ_42_ oligomers obtained after 8 h of incubation at a concentration of 10 µM in PBS, pH 7.4, 37 °C, were added to the culture medium of SH-SY5Y cells seeded on glass coverslips for 1 h at 0.5 µM. After incubation, the cells were washed with PBS, the plasma membranes were counterstained with 0.01 mg/ml tetramethylrhodamine-conjugated wheat germ agglutinin (WGA; Thermo Fisher Scientific) for 15 min at 37 °C and the cells were then fixed with 2.0% (w/v) paraformaldehyde for 10 min at RT, as reported^37,48^. After washing with PBS, the plasma membranes were permeabilized with a 3.0% (v/v) glycerol solution for 10 min at RT. Aβ_42_ species were then detected with 3 μM and 1 μM DesAb-O or DiDesAb-O and 1:800 6E10 Ab as a control. As a secondary Ab, we used diluted FITC anti-6X His-tag secondary Abs (Abcam) or AF488-conjugated anti-mouse secondary Abs (Thermo Fisher Scientific).

In another set of experiments, we calculate the number of Aβ_42_ oligomers bound to neuronal membranes of SH-SY5Y. To perform this experiment, 0.5 μM Aβ_42_ oligomers were pre-incubated or not with decreasing sdAbs molar ratios (1:3, 1:2, 1:1, 1:0.5, 1:0.25, and 1:0.1 corresponding to 7.5, 5, 2.5, 1.25, 0.63, and 0.25 µM of sdAbs concentrations) for 1 h at 37 °C under soft shaking, and cells were then treated for 15 min. At the end of treatment, cells were washed with PBS, and the plasma membranes were counterstained for 15 min with 5.0 μg mL^−1^ AF633-conjugated WGA (Thermo Fisher Scientific)^37^. Cells were fixed in 2.0% (w/v) paraformaldehyde for 10 min at RT, and Aβ_42_ assemblies were detected with 1:800 diluted 6E10 Ab, and then 1:1000 diluted AF488-conjugated anti-mouse secondary Ab (Thermo Fisher Scientific). To detect only the oligomers bound to the cell surface, the cellular membrane was not permeabilized, thus preventing Ab internalization. Fluorescence emission was detected after double excitation at 633 and 488 nm by a TCS SP8 scanning confocal microscopy system (Leica Microsystems)^47^. The number of oligomers was estimated in regions of interest in 60–80 cells, via the use of ImageJ software (NIH) and JACOP plugin (http:// rsb. info. nih. gov) (Rasband WR).

### Stimulated emission depletion (STED) microscopy

Synthetic Aβ_42_ aggregates collected at different timepoints (0, 2, 4, 8, and 24 h) were spotted on a glass coverslip for 30 min. Then, the samples were blocked with 1x casein for 30 min, washed with PBS, and incubated with 1:500 diluted 19.3, 1:800 diluted 6E10 or 1:800 diluted OC primary Abs for 1 h and 30 min. After washing with PBS, the samples were incubated with 1:500 diluted AF488-conjugated anti-human, AF514-conjugated anti-mouse and AF514-conjugated anti-rabbit secondary Abs. Fluoromount-G™ (Fisher Scientific) was used as mounting medium. STED xyz images (i.e., z-stacks were acquired at 0.1 μm intervals along 3 directions: x, y, and z axes) in bidirectional mode with a Leica SP8 STED 3X confocal microscope system equipped with a Leica HC PL APO CS2 100x/1.40 oil STED White objective^37^. AF514 was excited with a 510 nm-tuned WLL and emission collected from 532 to 551 nm. Frame sequential acquisition was applied to avoid fluorescence overlap. A gating between 0.3 to 6 ns to avoid collection of reflection and autofluorescence was applied. Six hundred fifty nanometers pulsed depletion laser was used for AF514 excitation. Collected images were de-convolved with Huygens Professional software version 18.04 (Scientific Volume Imaging B.V.) and analyzed with Leica Application Suite X (LAS X) software (Leica).

### Measurement of cytosolic free Ca^2+^ levels

The intracellular calcium levels were measured in SH-SY5Y cells^37,71^. SH-SY5Y cells were treated for 15 min with 0.5 μM synthetic Aβ_42_ oligomers previously incubated or not for 1 h at 37 °C under gentle shaking with decreasing sdAbs molar ratios (1:3, 1:2, 1:1, 1:0.5, 1:0.25, and 1:0.1 corresponding to 7.5, 5, 2.5, 1.25, 0.63, and 0.25 µM sdAb). Cells were then washed in PBS and loaded with 4.5 μM Fluo-4 AM (Thermo Fisher Scientific) for 10 min, and cytosolic Ca^2+^ levels were detected after excitation at 488 nm by the TCS SP8 scanning confocal microscopy system (Leica Microsystems, Mannheim, Germany), equipped with an argon laser source. For each sample, a series of 1-μm-thick optical sections (1024 × 1024 pixels) was acquired across the entire cell depth using a Leica Plan Apo 63× oil immersion objective. To generate a single representative view, these sections were merged into a composite maximum intensity projection. During acquisition, pinhole diameter, detector gain, and laser intensity were optimized and kept strictly constant across all experimental groups to ensure comparability. Image processing was performed using the ImageJ software (NIH, Bethesda, MD, USA). Fluorescence levels were quantified and are reported as a percentage relative to the untreated control cells^37,48,71^.

In another set of experiments, SH-SY5Y cells were treated for 5 h with the CSFs (*n* = 4 for AD as well as for controls) pre-incubated or not with DesAb-O or DiDesAb-O at 3 μM and 1 μM for 1 h at 37 °C under shaking^37^. Images were then analyzed using the ImageJ software, and the fluorescence intensities were expressed as the percentage of that measured in untreated cells, taken as 100%.

### MTT reduction assay

To assess the cytotoxicity of Aβ_42_ oligomers, a MTT reduction assay was conducted. Briefly, 8 h Aβ_42_ oligomers were pre-incubated for 1 h at 37 °C with decreasing sdAbs molar ratios (1:3, 1:2, 1:1, 1:0.5, 1:0.25, and 1:0.1 corresponding to 7.5, 5, 2.5, 1.25, 0.63, and 0.25 µM sdAbs) and then added to the extracellular medium of SH-SY5Y cells. After treatment, the cell culture medium was removed, the cells were washed in PBS, and the MTT solution was added to the cells for 4 h. The formazan product was solubilized with cell lysis buffer (20% sodium dodecyl sulfate (SDS), 50% N, N-dimethylformamide, pH 4.7) for 1 h. The absorbance values of blue formazan were determined at 595 nm. MTT tests were achieved using Microplate Manager® Software (Biorad). Cell viability was expressed as the percentage of MTT reduction relative to untreated cells (taken as 100%), or to those treated with Aβ_42_ species in the absence of sdAbs^37^.

In another set of experiments to assess the cytotoxicity of synthetic Aβ_42_ aggregates formed during the Aβ_42_ aggregation process, another MTT reduction assay was conducted. 1 µM Aβ_42_ species (monomer equivalents) collected from a ThT aggregation assay at different timepoints (0, 2, 4, 8, and 24 h) were added for 24 h to the culture medium of SH-SY5Y cells. 1 µM Aβ_42_ ADDLs were used as positive control. For many experiments, we used Aβ_42_ oligomers obtained after 8 h of incubation at 37 °C. We also performed an MTT test with decreasing concentrations of 8 h Aβ_42_ oligomers (1 µM, 0.5 µM, 0.25 µM, 1 nM, 0.5 nM, 0.25 nM, and 1 pM) to optimize our experimental conditions. MTT tests were performed as reported in the main text.

### Statistical analysis and reproducibility

Data were expressed as means ± standard deviation (S.D.), or as means ± standard error of mean (S.E.M.), as indicated in each figure legend. Comparisons between the different groups were performed by using one-tailed Student *t* test or by one-way or two-way ANOVA followed by Bonferroni’s multiple-comparison test (GraphPad Prism 10.3.1 software), as indicated in each figure legend. *P* values lower than 0.05, 0.01, 0.001, and 0.0001 were considered to be statistically significant (*), highly statistically significant (**), very highly statistically significant (***), and extremely highly statistically significant (****), respectively. Sample sizes (*n*) are specified in the respective figure legends. For experiments involving human CSF, samples were obtained by pooling equal volumes from either non-demented aged controls (*n* = 4) or AD patients (*n* = 4). No data were excluded from the analysis. In real-time ELISA-based assays and cell biology measurements, treatments were distributed across multi-well plates using random allocation. Furthermore, investigators were blinded to clinical information and experimental groups during data collection and analysis.

### Reporting summary

Further information on research design is available in the Nature Portfolio Reporting Summary linked to this article.

## Supplementary information

Supplementary Information.pdf

Reporting Summary

Transparent Peer Review File

## Data Availability

All data have been made freely available. The complete datasets containing all experimental values are accessible on Zenodo: 10.5281/zenodo.18339814^72^. Data are provided in a multi-sheet Excel format, with individual sheets corresponding to the different experimental conditions and parameters for each figure described in this study. Uncropped scans of membranes, dot blots and gels are provided in the Supplementary Information. All other data are available from the corresponding author (or other sources, as applicable) on reasonable request.
