# Supplementary material for: Engineering a dimeric single-domain antibody for improved detection and neutralization of amyloid-β oligomers
Source: Commun Biol. 2026 Mar 27;9:896. doi: 10.1038/s42003-026-09740-6 (PMC13332026; doi:10.1038/s42003-026-09740-6)
Supplement: Supplementary file 1 — Supplementary Information.pdf [file 42003_2026_9740_MOESM1_ESM.pdf]

Supplementary figures and legends

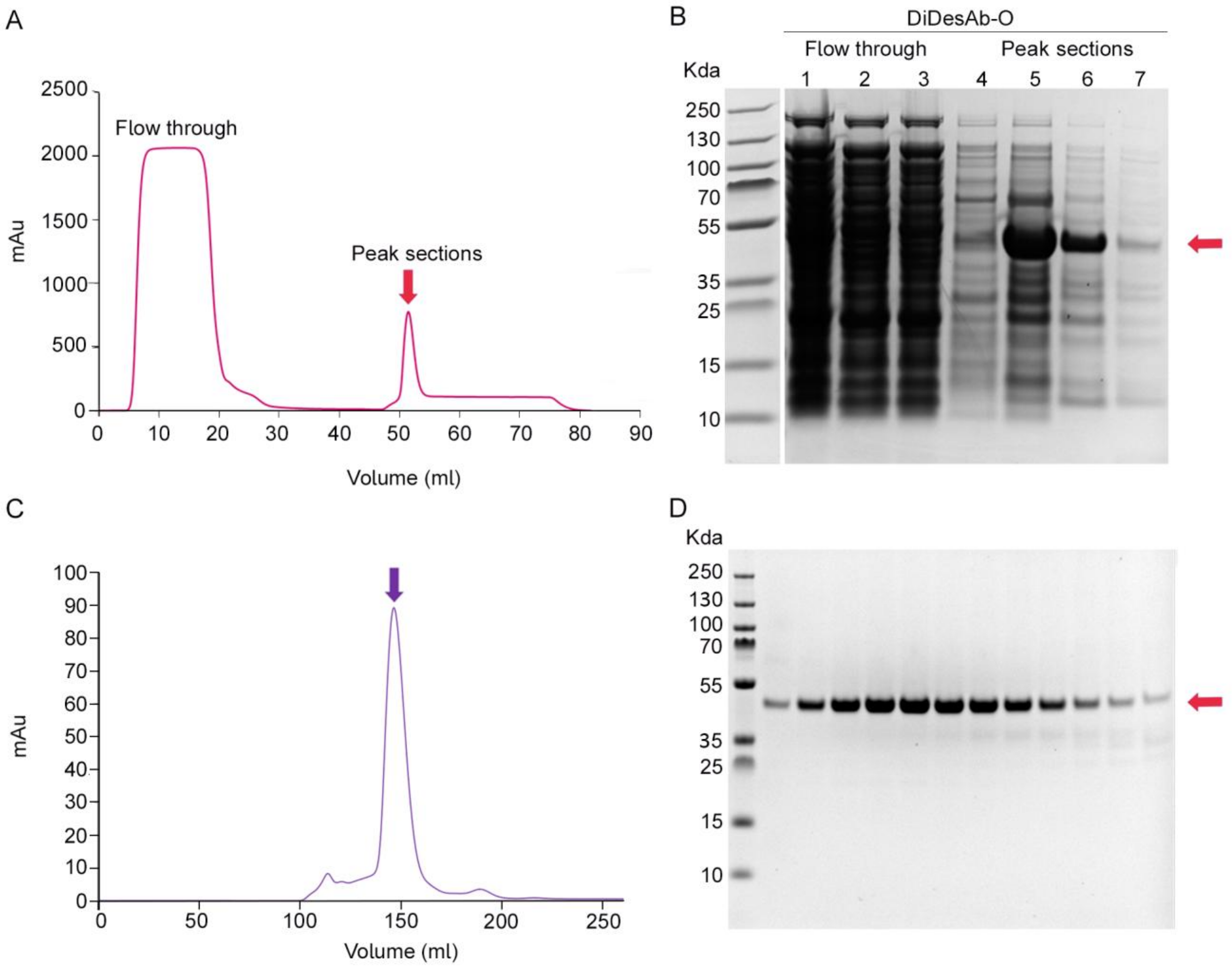

Supplementary Figure 1. DiDesAb-O expression and purification. A Ni^{2+}-NTA chromatogram of lysed *E. coli* supernatant containing DiDesAb-O (red arrow). **B** SDS-PAGE of samples taken after Ni^{2+}-NTA chromatography; flow through (lanes 1-3), peak fractions (lane 4-7). DiDesAb-O molecular weight is indicated by the red arrow. Full uncropped and unedited scan of the gel is provided in Supplementary Figure 8C. **C** SEC chromatogram of the sample fractions after Ni^{2+}-NTA chromatography and containing DiDesAb-O. The peak relative to DiDesAb-O is indicated by the purple arrow. **D** SDS-PAGE of DiDesAb-O peak fractions taken after SEC to verify the sample purity. DiDesAb-O molecular weight is indicated by the red arrow. Full uncropped and unedited scan of the gel is provided in Supplementary Figure 8C.

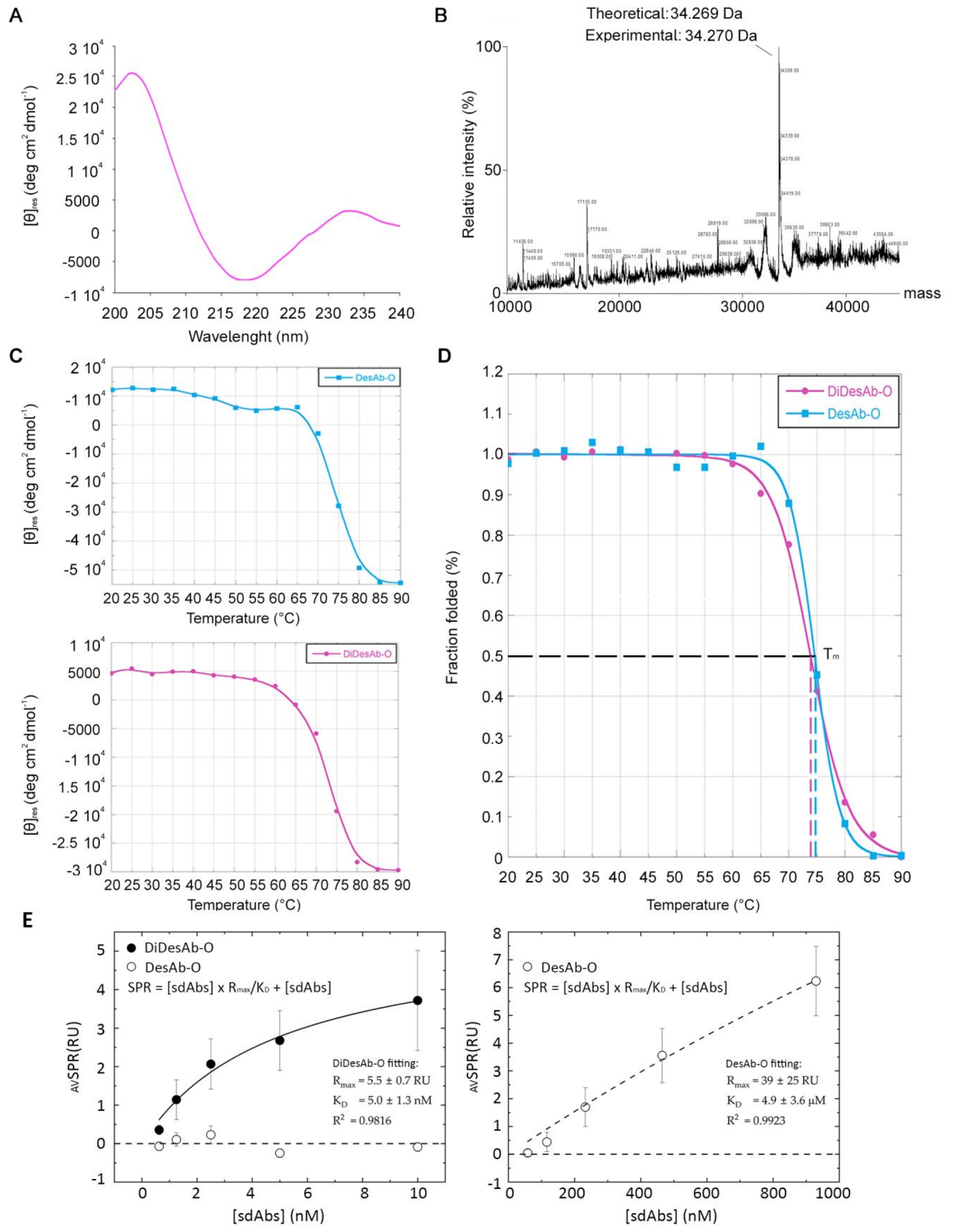

Supplementary Figure 2. **Structural characterization of DiDesAb-O. A** Far-UV CD spectrum of DiDesAb-O. The presence of peaks at 202 nm and 218 nm highlights a predominant β-sheet secondary structure. **B** ESI-MS spectrum. Theoretical molecular weight was determined by Web tools such as ExPASy ProtParam tool (34.269 Da) being perfectly in line with the mass observed by ESI-MS (34.270 Da). **C** DesAb-O (top) and DiDesAb-O (bottom) denaturation curves were obtained using $[\theta]_{210}$ plotted against temperature between 20 °C and 90 °C. **D** DesAb-O and DiDesAb-O denaturation curves fitted with the Santoro and Bolen equation and normalized to fraction folded (%)

values. DiDesAb-O had a temperature of half-denaturation (T_m) of 73.7 °C, while the value for DesAb-O was 74.6 °C. **E** SPR analysis of the interaction of DesAb-O (empty circles) and DiDesAb-O (filled circles) with ADDLs using single-cycle kinetics (SCK) injections. Binding curves were recorded in real time at 37 °C in PBS (pH 7.4). Circles represent the average binding responses at a defined stability time point along the recorded curves from three repeated measurements. Error bars indicate standard deviations. Both analytes were injected over $A\beta_{42}$ ADDLs covalently immobilized on the CM5 sensor chip surface. sdAbs concentrations ranged from 0.625 to 10.0 nM (left) or from 51 to 930 nM for the monomer (right). The response units (RU) reflect the real-time binding to the immobilized ligand, with higher signals indicating stronger interactions. Apparent K_D, RU_{max}, and R^2 values were calculated from the average binding responses.

	DesAb-O (%)	DiDesAb-O (%)
**Helix 1 (regular)**	0.0	0.0
**Helix 2 (distorted)**	0.0	2.6
**Anti-parallel 1 (left-twisted)**	6.4	8.8
**Anti-parallel 2 (relaxed)**	34.7	29.9
**Anti-parallel 3 (right-twisted)**	15.5	21.9
**Parallel**	6.9	0.0
**Turn**	8.8	10.4
**Others**	27.7	26.3

Supplementary Table 1. Estimated secondary structure content (%) obtained with BestSel software[1] (v1.3.230210) between the wavelength range of 195 and 250.

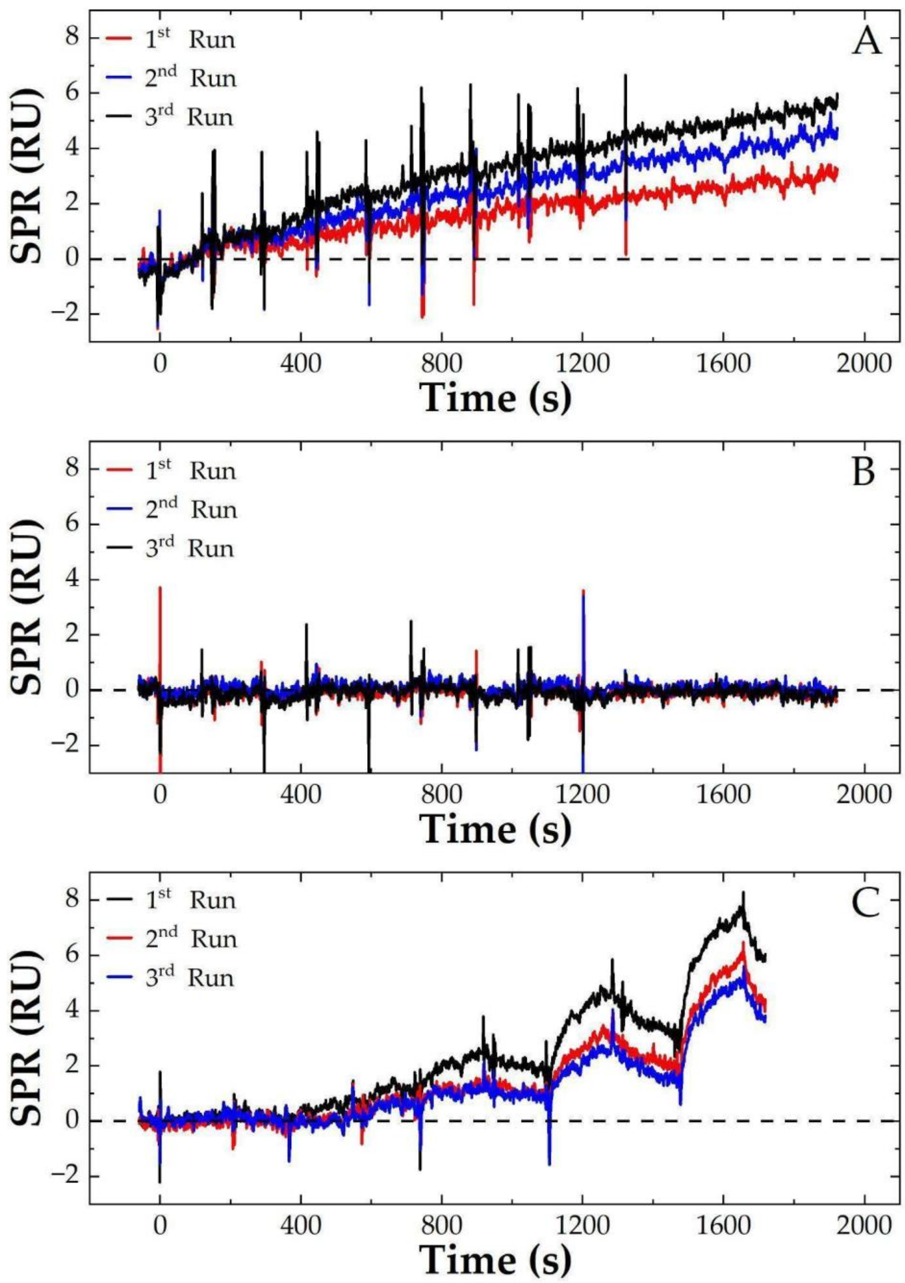

Supplementary Figure 3. Raw sensograms from SPR analysis of the sdAbs–ADDLs interaction using the single-cycle (SCK) method. DiDesAb-O (A) or DesAb-O (B, C) was injected over ADDLs covalently immobilized on the CM5 sensor chip surface. Sensorgrams were recorded in real time at 37 °C in PBS (pH 7.4) in triplicate. SdAbs concentrations ranged from 0.625 to 10.0 nM for the dimer (A) and monomer (B), and from 51 to 930 nM for the monomer (C).

[DiDesAb-O] (nM)

**[DiDesAb-O] (nM)**	**$_{av}$SPR (RU)***	**SD (RU)**
0.625	0.35	0.12
1.25	1.14	0.52
2.5	2.07	0.65
5.0	2.68	0.78
10.0	3.72	1.30
**[DesAb-O] (nM)**	**$_{av}$SPR (RU)***	**SD (RU)**
0.625	-0.07	0.11
1.25	0.10	0.17
2.5	0.23	0.23
5.0	-0.25	0.11
10.0	-0.09	0.11
**[DesAb-O] (nM)**	**$_{av}$SPR (RU)***	**SD (RU)**
58.125	0.05	0.12
116.25	0.44	0.35
232.5	1.69	0.70
465.0	3.55	0.97
930.0	6.23	1.25

*Values represent the average of three measurements at a defined stability time point along the real-time sensorgrams recorded at 37 °C in PBS (pH 7.4). Analytes were injected over ADDLs covalently immobilized on the CM5 sensor chip surface.

Supplementary Table 2. SPR analysis of the sdAbs–ADDLs interaction using single-cycle (SCK) injections.

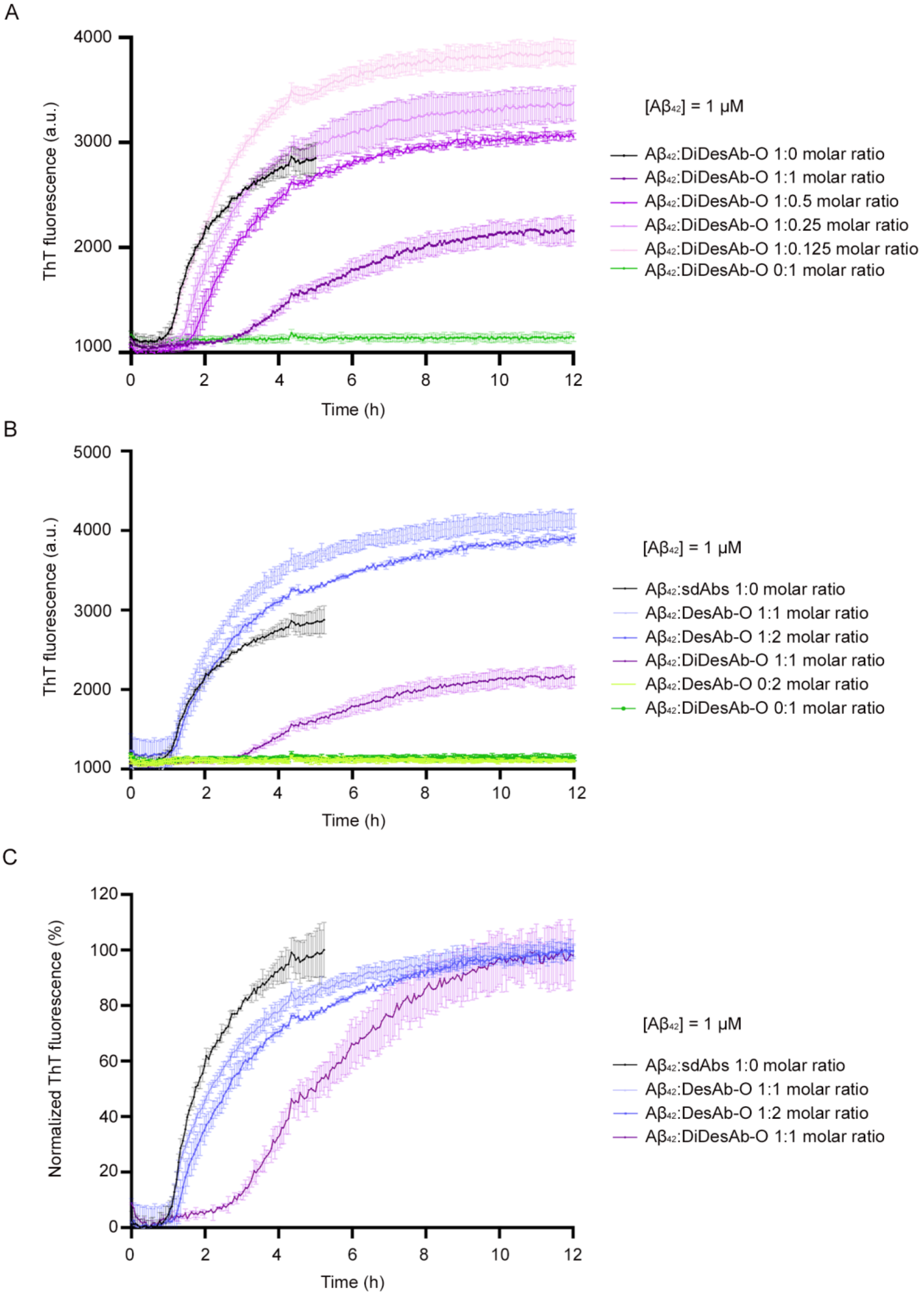

Supplementary Figure 4. Raw data of the ThT aggregation assays illustrated in Figure 2. **A,B** Raw ThT aggregation data for 1 µM Aβ_{42} aggregations at varying Aβ_{42}:DiDesAb-O **(A)** and Aβ_{42}:DesAb-O **(B)** ratios. Controls included DiDesAb-O and DesAb-O alone (1 µM and 2 µM, respectively). Two or three replicates were averaged per condition. **C** Normalization of the ThT assay kinetic traces represented in **B**. In all panels, error bars refer to standard deviations.

Aβ$_{42}$:DiDesAb-O molar ratios	t_{50} (h)
**1:1**	3.2 ± 1.1
**1:0.5**	2.0 ± 0.6
**1:0.25**	1.8 ± 0.5
**1:0.125**	1.7 ± 0.5
**1:0**	1.5 ± 0.4

Supplementary Table 3. t_{50} (h) of solutions containing ThT and decreasing Aβ$_{42}$:DiDesAb-O molar ratios.

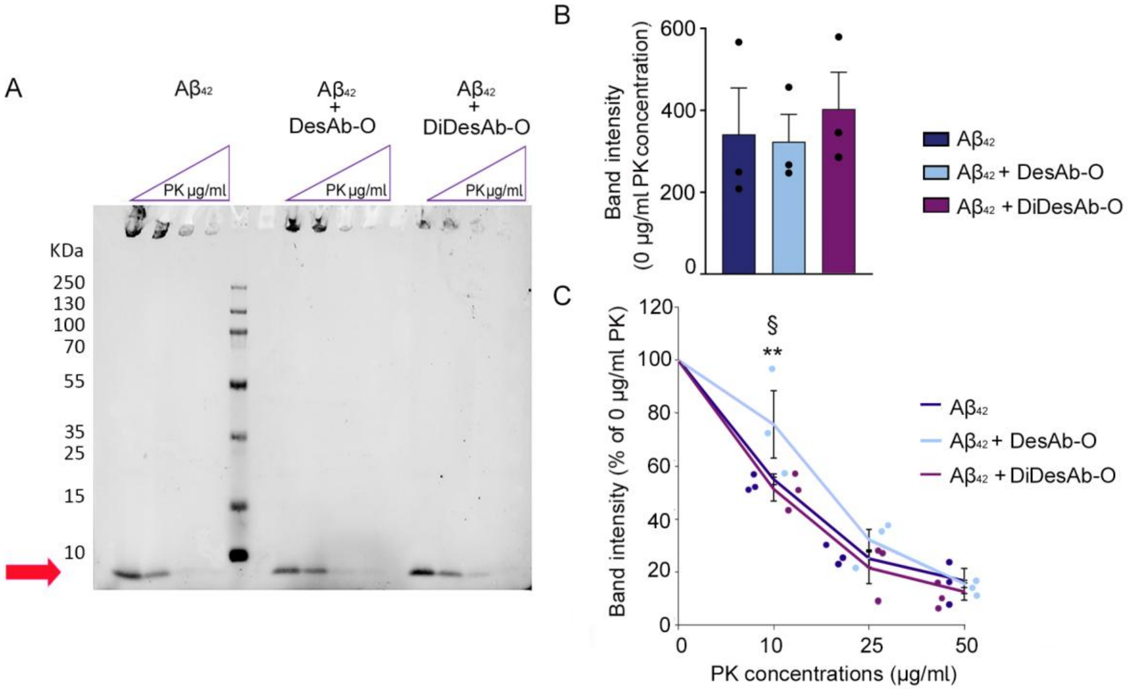

Supplementary Figure 5. Evaluation of PK cleavage sensitivity in Aβ$_{42}$ fibrils obtained in absence or presence of sdAbs. A Representative Western Blot of Aβ$_{42}$ fibrils obtained after 4 days at 37 °C with or without co-incubation with sdAbs, treated with increasing PK concentrations (0, 10, 25, 50 μg/mL) for 30 mins. Full uncropped and unedited scan of the membrane is provided in Supplementary Figure 8D. **B** Quantification of 0 μg/mL band intensity (n = 3) for each treatment. **C** Evaluation of Aβ$_{42}$ fibril resistance to the PK digestion at increasing PK concentrations (0, 10, 25, 50 μg/mL). Experimental errors are S.E.M. Samples (n = 3) were analyzed by two-way ANOVA followed by Bonferroni's multiple-comparison test relative to Aβ fibrils obtained without sdAb versus Aβ fibrils obtained in the presence of DesAb-O (§ $P < 0.05$), or to Aβ fibrils obtained in the presence of DiDesAb-O compared to Aβ aggregates obtained in the presence of DesAb-O (** $P < 0.01$).

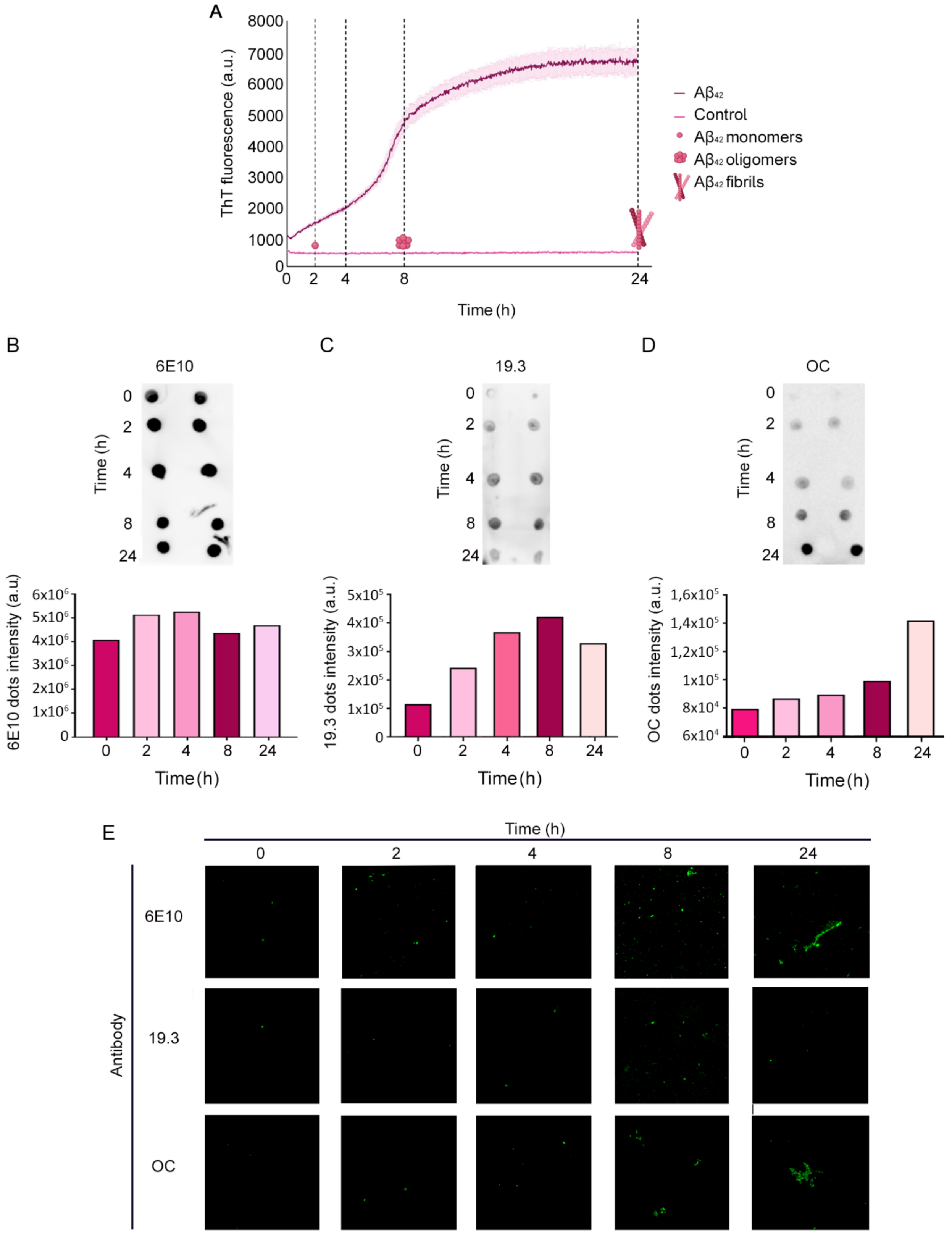

Supplementary Figure 6. Characterisation of Aβ_{42} aggregates starting from the synthetic peptide. A Monomeric Aβ_{42} was incubated in PBS at 10 µM with 25 µM ThT dye, 37 °C. Time points were collected at 0, 2, 4, 8 and 24 h to perform a characterization of Aβ_{42} aggregates. **B-D** Dot blot analysis and quantification of Aβ_{42} samples collected at different timepoints (n = 2 technical replicates). Samples of different Aβ_{42} species were deposited (2 µl/spot) onto a nitrocellulose membrane and detected with the indicated antibodies (Abs). Membranes were incubated with 6E10 (**B**), 19.3 (**C**) and OC (**D**) primary Abs. Full uncropped and unedited scans of the Dot Blots are provided in Supplementary Figure 8E. **E)** STED microscopy images and visualisation of Aβ_{42} samples collected at different timepoints. Samples of

different Aβ$_{42}$ species were immunolabeled with 6E10, 19.3 and OC Abs. The STED microscopy images are perfectly in line with the dot blot assay results, showing the presence of oligomers since the initial stage of the aggregation Aβ$_{42}$ process as represented in 0, 2 and 4 h of 19.3 Abs images. After 24 h, the presence of fibrils is proved by the OC Abs signal confirming the aggregation assay and dot blot assay results.

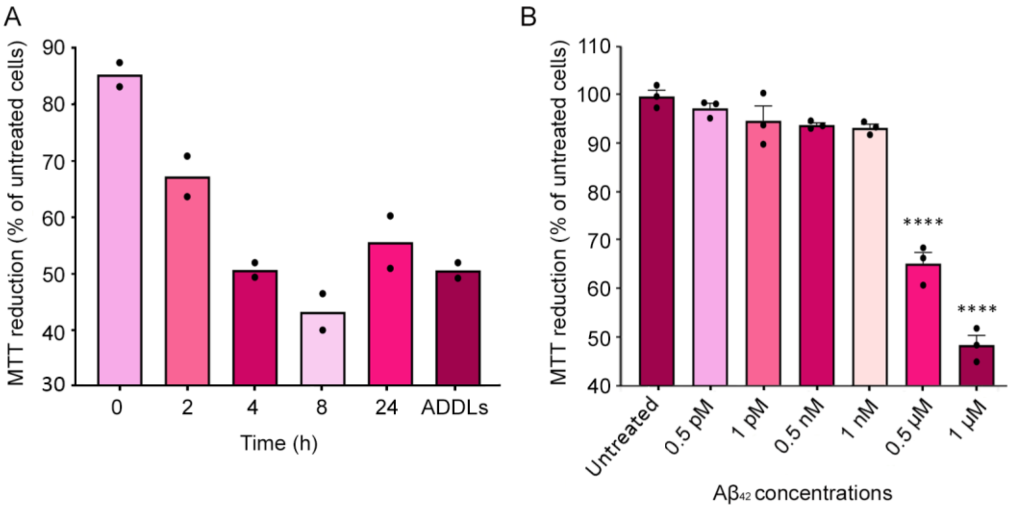

Supplementary Figure 7. **Aβ$_{42}$ oligomeric species obtained after 8 h of incubation exhibit high toxicity. A** MTT reduction in SH-SY5Y cells treated for 24 h with various Aβ$_{42}$ aggregates (1 µM) collected at different timepoints (0, 2, 4, 8 and 24 h) during aggregation (n = 2). ADDLs were used as a positive control. The most toxic aggregates were obtained after 8 h of aggregation, being in line with previous evidence of high Aβ$_{42}$ oligomers concentration at this timepoint (**Supplementary Figure 6**). **B** MTT reduction in SH-SY5Y cells treated for 24 h with Aβ$_{42}$ aggregates obtained after 8 h of aggregation in a dose-dependent manner. Experimental errors are S.E.M. Samples (n = 3 for B) were analysed by One-way ANOVA followed by Bonferroni's multiple comparison test relative to untreated cells (****P<0.0001).

Supplementary Figure 8

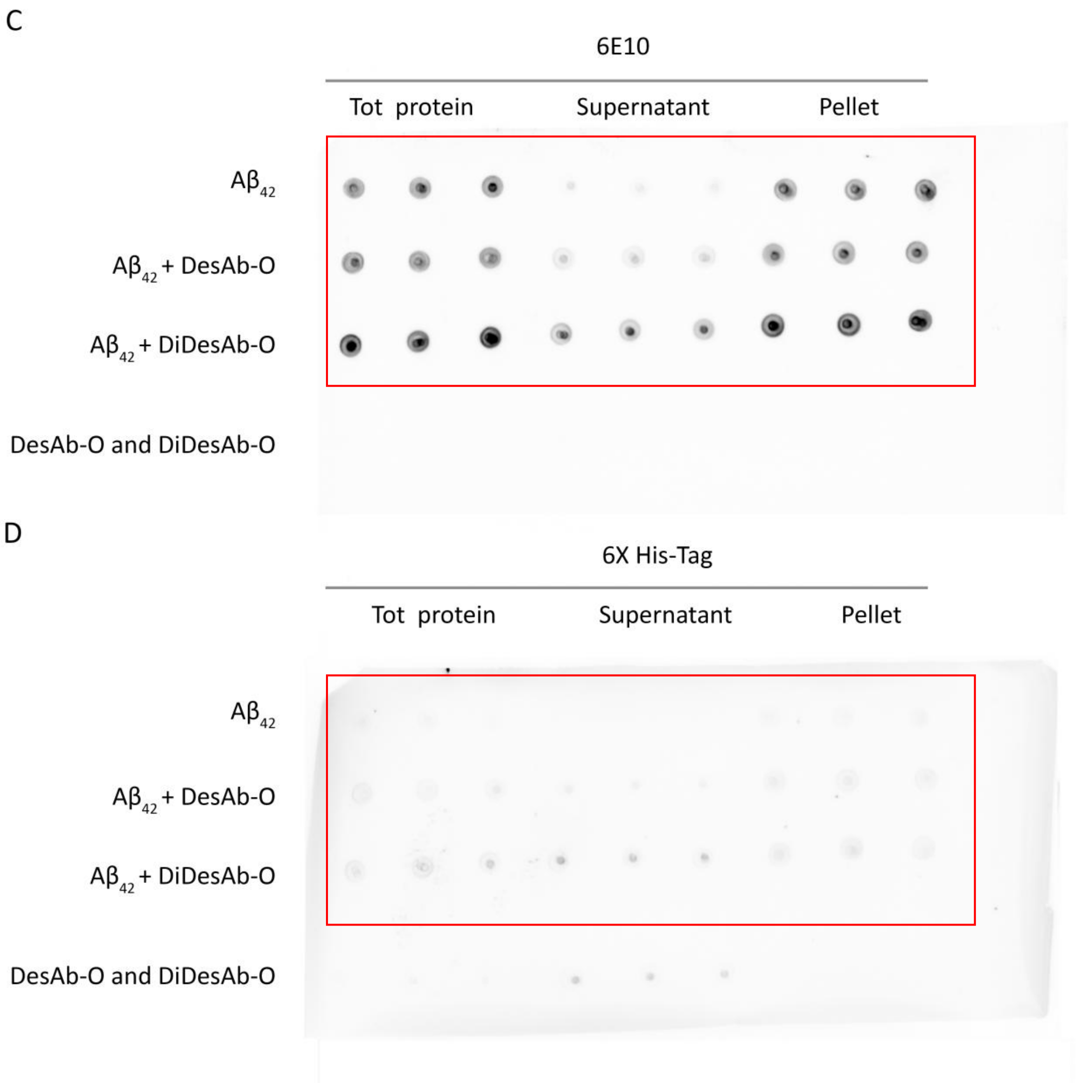

Supplementary Figure 8A. Original scans for Dot Blots in Figure 3. Full unedited membrane for Dot Blot shown in Figure 3C and Figure 3D. The red boxes indicate the cropped areas used in the final figure

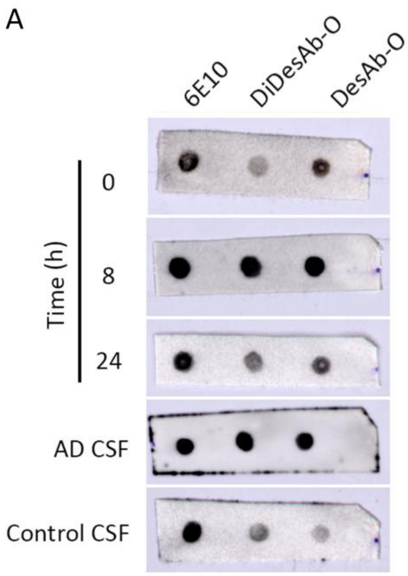

Supplementary Figure 8B. Original scans for Dot Blots in Figure 7. Full unedited membrane for Dot Blot shown in Figure 7A.

B

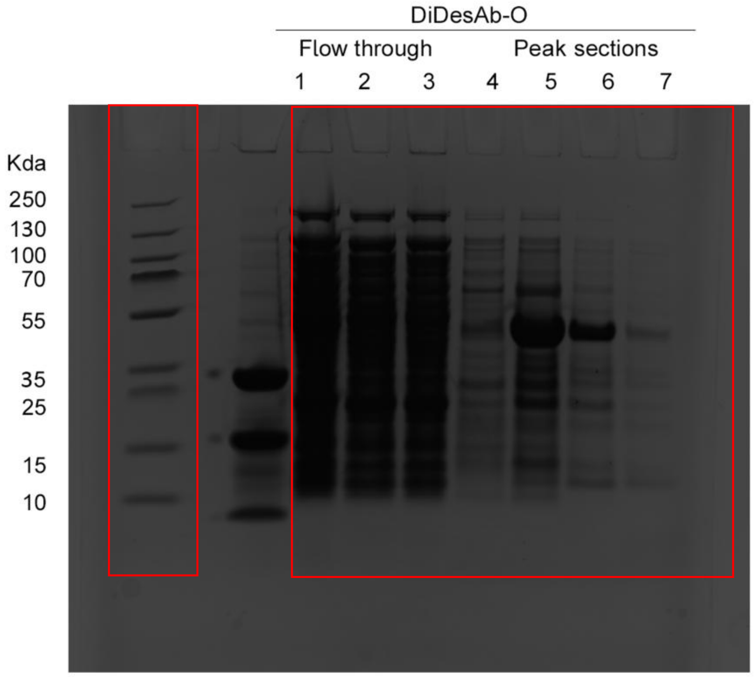

D

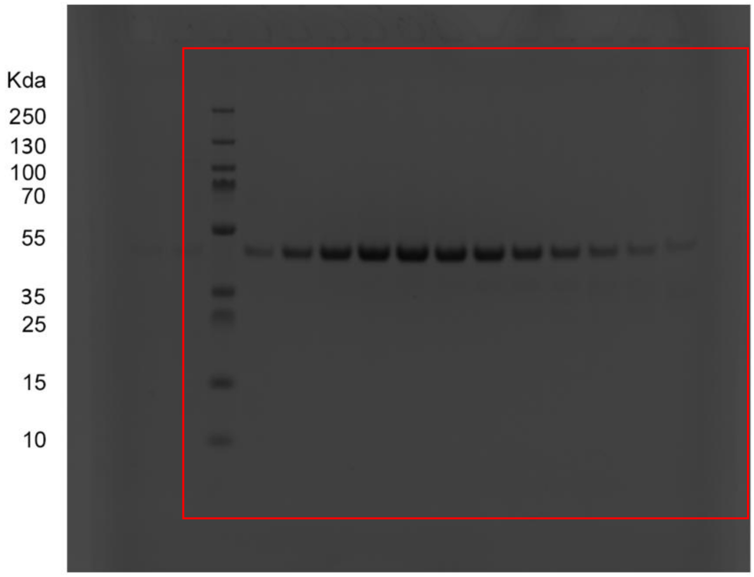

Supplementary Figure 8C. Original scans for gels shown in Supplementary Figure 1B and D. Red boxes indicate the cropped areas used in the final figure.

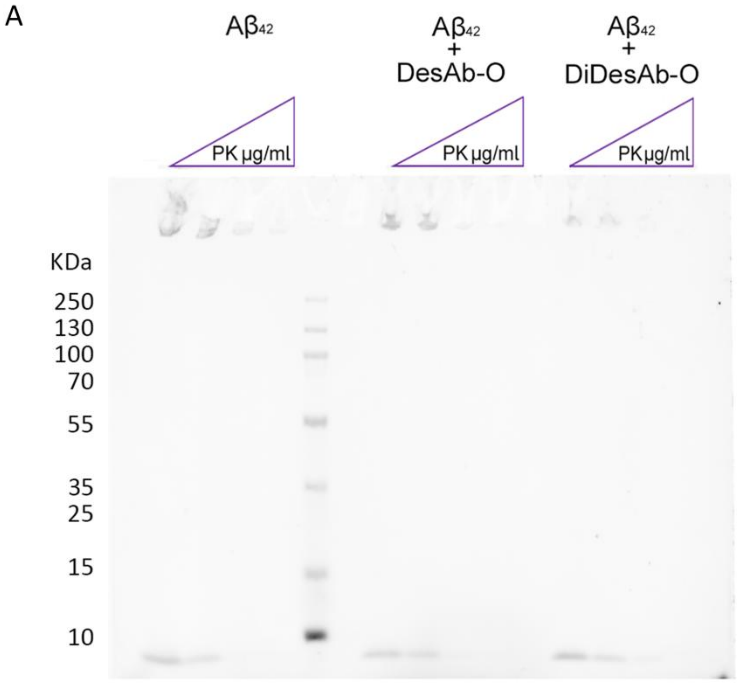

Supplementary Figure 8D. Original scans for Western Blot in Supplementary Figure 5.

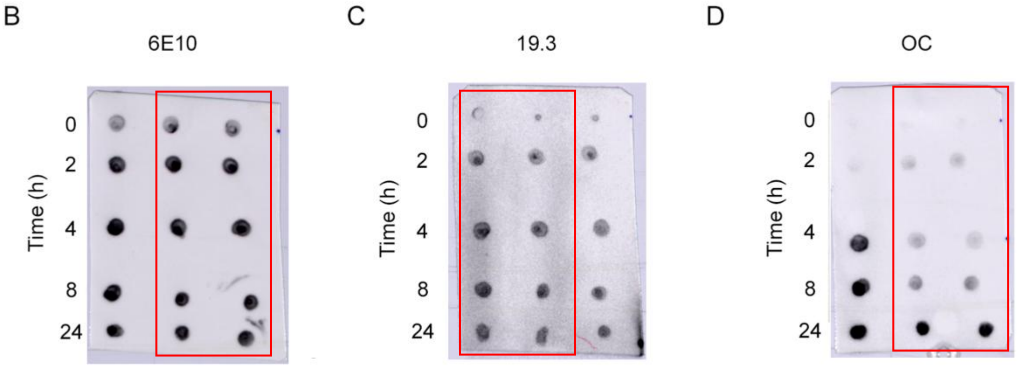

Supplementary Figure 8E. Original scans for Dot Blots in Supplementary Figure 6. Full unedited membrane for Dot Blot shown in Supplementary Figure 6B, Supplementary Figure 6C and Supplementary Figure 6D. The red boxes indicate the cropped areas used in the final figure

Supplementary References

1. Micsonai, A. *et al.* BeStSel: a web server for accurate protein secondary structure prediction and fold recognition from the circular dichroism spectra. *Nucleic Acids Res.* **46**, W315–W322 (2018).
