# Supplementary material for: Engineering a dimeric single-domain antibody for improved detection and neutralization of amyloid-β oligomers
Source: Commun Biol. 2026 Mar 27;9:896. doi: 10.1038/s42003-026-09740-6 (PMC13332026; doi:10.1038/s42003-026-09740-6)
Supplement: Supplementary file 2 — Reporting Summary [file 42003_2026_9740_MOESM2_ESM.pdf]

nature portfolio

Corresponding author(s): Roberta Cascella and Francesco A. Aprile

Last updated by author(s): 2026/01/23

Reporting Summary

Nature Portfolio wishes to improve the reproducibility of the work that we publish. This form provides structure for consistency and transparency in reporting. For further information on Nature Portfolio policies, see our Editorial Policies and the Editorial Policy Checklist.

Statistics

For all statistical analyses, confirm that the following items are present in the figure legend, table legend, main text, or Methods section.

n/a	Confirmed	
☐	☒	The exact sample size (*n*) for each experimental group/condition, given as a discrete number and unit of measurement
☐	☒	A statement on whether measurements were taken from distinct samples or whether the same sample was measured repeatedly
☐	☒	The statistical test(s) used AND whether they are one- or two-sided<br>*Only common tests should be described solely by name; describe more complex techniques in the Methods section.*
☒	☐	A description of all covariates tested
☐	☒	A description of any assumptions or corrections, such as tests of normality and adjustment for multiple comparisons
☐	☒	A full description of the statistical parameters including central tendency (e.g. means) or other basic estimates (e.g. regression coefficient) AND variation (e.g. standard deviation) or associated estimates of uncertainty (e.g. confidence intervals)
☒	☐	For null hypothesis testing, the test statistic (e.g. *F*, *t*, *r*) with confidence intervals, effect sizes, degrees of freedom and *P* value noted<br>*Give P values as exact values whenever suitable.*
☒	☐	For Bayesian analysis, information on the choice of priors and Markov chain Monte Carlo settings
☒	☐	For hierarchical and complex designs, identification of the appropriate level for tests and full reporting of outcomes
☐	☒	Estimates of effect sizes (e.g. Cohen's *d*, Pearson's *r*), indicating how they were calculated

Our web collection on statistics for biologists contains articles on many of the points above.

Software and code

Policy information about availability of computer code

Data collection	Protein purification chromatograms were generated using an ÄKTA Pure system (Cytiva), which was controlled by UNICORN software. Far-UV CD spectra of the proteins were acquired using a Chirascan spectropolarimeter (Applied Photophysics). Denaturation curves were fitted using the Santoro and Bolen equation with Kaleidagraph software (v. 4.0). For Western blot and dot blot imaging, fluorescence signals were captured using a Typhoon FLA 9500 controlled by Image Lab software (version 13.0.6). To characterise toxic Aβ oligomers using different conformational primary antibodies, dot blots were detected using SuperSignal West Dura (Pierce) and ImageQuant TL software (version 8.2, GE Healthcare UK Limited). Thioflavin T fluorescence assays were performed using a BioTek Synergy H1 hybrid multi-mode reader (Agilent Technologies) to obatin toxic Aβ oligomers, and a ClarioStar Plus microplate reader (BMG Labtech) was used for aggregation assays in the presence or absence of sdAbs and the samples collection for real-time based Elisa assays. The results of real-time based ELISA assays were quantified using a ClarioStar plate reader (BMG Labtech). Transmission electron microscopy grids were imaged on a T12 Spirit electron microscope (Thermo Fisher Scientific), and the diameters of the fibrils were measured using ImageJ software. All data were plotted using Excel (version 16.89.1). Confocal microscopy images were acquired using Leica Application Suite Advanced Fluorescence software (Leica Microsystems, Mannheim, Germany), and STED images were acquired using Leica Application Suite X software (Leica). MTT tests were archived using Microplate Manager® software (Bio-Rad, CA, USA). Surface Plasmon Resonance (SPR) were performed on a Biacore X100 instrument (Cytiva, Global Life Science Solutions, Marlborough, USA).
Data analysis	The protein sequences were designed using SnapGene software, and specific regions of the protein sequences were identified using Jalview software (v. 2.11.40). The statistics associated with the experiments were determined using GraphPad Prism 10.3.1, all using the tests as described in the methods section of the manuscript. The SPR data analysis and graphing were performed using OriginPro (Version 2025, OriginLab Corporation, Northampton, MA, USA).

For manuscripts utilizing custom algorithms or software that are central to the research but not yet described in published literature, software must be made available to editors and reviewers. We strongly encourage code deposition in a community repository (e.g. GitHub). See the Nature Portfolio guidelines for submitting code & software for further information.

Data

Policy information about availability of data

All manuscripts must include a data availability statement. This statement should provide the following information, where applicable:

- Accession codes, unique identifiers, or web links for publicly available datasets
- A description of any restrictions on data availability
- For clinical datasets or third party data, please ensure that the statement adheres to our policy

The manuscript includes a data availability statement with all the required information

Research involving human participants, their data, or biological material

Policy information about studies with human participants or human data. See also policy information about sex, gender (identity/presentation), and sexual orientation and race, ethnicity and racism.

Reporting on sex and gender	Sex and gender were not considered in the study design. The cerebrospinal fluid used in this study arises from AD patients and age-matched non-demented individuals, selected independently of their sex and gender.
Reporting on race, ethnicity, or other socially relevant groupings	Nothing to report
Population characteristics	Human CSF samples from a commercial source were utilized
Recruitment	CSF samples from human aged controls (n=4) or AD (n=4) samples were purchased from BioVT (US)
Ethics oversight	Since commercial samples were used without identifying sensitive information, this work can be considered as a "non human subject research".

Note that full information on the approval of the study protocol must also be provided in the manuscript.

Field-specific reporting

Please select the one below that is the best fit for your research. If you are not sure, read the appropriate sections before making your selection.

☒ Life sciences ☐ Behavioural & social sciences ☐ Ecological, evolutionary & environmental sciences

For a reference copy of the document with all sections, see nature.com/documents/nr-reporting-summary-flat.pdf

Life sciences study design

All studies must disclose on these points even when the disclosure is negative.

Sample size	The sample size (n) is described in each figure legend. For the CSF experiments, a single pool of human cerebrospinal fluid was used, arising from non-demented aged controls or AD subjects.
Data exclusions	No data was excluded from the study
Replication	The sample size (n) is described in each figure legend
Randomization	In real-time based ELISA assay and Cell biology measurements, treatments were distributed throughout the multiwell plate using random allocation.
Blinding	The investigators who performed the experiments were blinded to clinical information.

Reporting for specific materials, systems and methods

We require information from authors about some types of materials, experimental systems and methods used in many studies. Here, indicate whether each material, system or method listed is relevant to your study. If you are not sure if a list item applies to your research, read the appropriate section before selecting a response.

Materials & experimental systems

n/a	Involved in the study
☐	☒ Antibodies
☐	☒ Eukaryotic cell lines
☒	☐ Palaeontology and archaeology
☒	☐ Animals and other organisms
☒	☐ Clinical data
☒	☐ Dual use research of concern
☒	☐ Plants

Methods

n/a	Involved in the study
☒	☐ ChIP-seq
☒	☐ Flow cytometry
☒	☐ MRI-based neuroimaging

Antibodies

Antibodies used

Mouse monoclonal antibody anti-Aβ (6E10) Ab (Biolegend), Anti-6X His-Tag Ab (Abcam), human monocloanl anti-ADDLs (19.3) ab (Creative Biolabs), Rabbit anti-amyloid fibrils (OC) Ab (Sigma-Aldrich), Goat anti-human conjugated with horseradish peroxidase (HRP) (Sigma-Aldrich), Rabbit anti-mouse HRP-conjugated (Abcam), Goat anti-rabbit HRP-conjugated (Abcam), Alexa Fluor 647-conjugated secondary Ab (Thermo Fisher scientific), Alexa Fluor 488-conjugated anti-mouse (Thermo Fisher Scientific), Alexa Fluor 488-conjugated anti-human, Alexa Fluor 514-conjugated anti-mouse and Alexa Fluor 514-conjugated anti-rabbit secondary Ab (Thermo Fisher Scientific). DesAb-O (anti- Aβ oligomers, rationally designed sdAb by Aprile et al., 2020, not commercially available), DiDesAb-O (anti-Aβ oligomers, obtained in this study, not commerically available).

Validation

The antibodies used in this study were validated from the above mentioned commercial sources. DesAb-O was tested in previous works (Aprile et al., 2020 and Bigi&Napolitano et al., 2024).

Eukaryotic cell lines

Policy information about cell lines and Sex and Gender in Research

Cell line source(s)

Human SH-SY5Y cells were acquired from A.T.C.C..

Authentication

Human SY5Y cell line was authenticated by the European Collection of Authenticated Cell Cultures using short tandem repeat loci analyses.

Mycoplasma contamination

The cells lines were tested negative for mycoplasm contamination

Commonly misidentified lines
(See ICLAC register)

Name any commonly misidentified cell lines used in the study and provide a rationale for their use.

Plants

Seed stocks

Report on the source of all seed stocks or other plant material used. If applicable, state the seed stock centre and catalogue number. If plant specimens were collected from the field, describe the collection location, date and sampling procedures.

Novel plant genotypes

Describe the methods by which all novel plant genotypes were produced. This includes those generated by transgenic approaches, gene editing, chemical/radiation-based mutagenesis and hybridization. For transgenic lines, describe the transformation method, the number of independent lines analyzed and the generation upon which experiments were performed. For gene-edited lines, describe the editor used, the endogenous sequence targeted for editing, the targeting guide RNA sequence (if applicable) and how the editor was applied.

Authentication

Describe any authentication procedures for each seed stock used or novel genotype generated. Describe any experiments used to assess the effect of a mutation and, where applicable, how potential secondary effects (e.g. second site T-DNA insertions, mosiacism, off-target gene editing) were examined.
