# Supplementary material for: Engineering a dimeric single-domain antibody for improved detection and neutralization of amyloid-β oligomers
Source: Commun Biol. 2026 Mar 27;9:896. doi: 10.1038/s42003-026-09740-6 (PMC13332026; doi:10.1038/s42003-026-09740-6)
Supplement: Supplementary file 3 — Transparent Peer Review File [file 42003_2026_9740_MOESM3_ESM.pdf]

nature portfolio

Peer Review File

Engineering a dimeric single-domain antibody for improved detection and neutralization of amyloid-β oligomers

Corresponding Author: Professor Roberta Cascella

This file contains all reviewer reports in order by version, followed by all author rebuttals in order by version.

Version 0:

Reviewer comments:

Reviewer #1

(Remarks to the Author)
This article is part of intensive research into Alzheimer's disease (AD), with a particular focus on soluble Aβ42 oligomers, identified as the most neurotoxic entities. The need for detection probes and therapeutic agents specific to these oligomers is crucial, given their pathogenic role and the difficulty of targeting them specifically.
The authors aim to improve the binding properties of an existing VHH or nanobody, DesAb-O, specific for Aβ42 oligomers. They designed a dimeric version, DiDesAb-O, to increase sensitivity and efficacy through an avidity effect. The design of dimeric variants has been widely used over the past decade, mainly in virology, to increase neutralising activity via an increase in avidity.
The authors have carried out an in-depth analysis of the dimeric variant: structure, effect on fibrils and prevention of toxicity. All these results are interesting but not really remarkable.

However, in the final section, the authors analysed the neutralising role of the dimer on the toxicity of oligomers present in the CSF of AD patients. They showed that the dimer is much more neutralising than the monomer.

This study is well documented and i is interesting to raise a dimer to improve the properties of the monomer against AD oligomers.

I consider it worthy of publication in Communications Biology

Reviewer #2

(Remarks to the Author)
The authors started with their improved designed singel domain antibody referred to as DesAb-O that binds to the AA stretch 29-36 of Aβ42. This DesAb-O (Aprile et al, 2020) had a relative weak affinity for Aβ42 oligomersand binds in a ratio of 1/5 (DesAb-O to Aβ42).
It is well established that the affinity of a weak recombinant binder can be approved by generating tandem repeats of this binder. Due to avidity effects such a dimerised binder will associate with its antigen (probably containing multiple epitopes) with a ≈100 fold lower KD value.
Hence the authors constructed a dimerised form of their DesAb-O joined by the standard (G4S)3 linkers and referred to this dimerised binder as DiDesAb-O. Consulting figure S1, providing the AA of the DiDesAb-O it appears that the construct also has a 36 amino acid leader sequence containing the His6 purification tag and downstream of the (G4S)3 linker a similar leder sequence has been introduced (but lacking the His6 tag). The reason to have this oligopeptide leader sequence or its origin are not mentioned. Thus what about the possible immunogenicity of these peptides? and what about the extravasation when such DiDesAb-O would be administered intravenously (see Debie et al, J. Control Release, 2020), and what about the BBB crossing? As long as in vitro experiments or on cell lines are performed you will not see any adverse effects but in vivo (organisms) these linkers will certainly have a negative effect, and thus prevent any subsequent translational efforts.
The authors compared the effect of this DiDesAb-O relative to the DesAb-O for binding to Aβ42 conformers and the possible neutralisation of their toxicity. Strangely enough no attempts have been made to measure the apparent KD values or the binding stoichiometry. The results seem to confirm that the DiDesAb-O performs better than the monomeric DesAb-O, as expected. However, one might wonder whether these statements are justified. The comparison were made using a fixed Aβ42 concentration (1 μM) and adding DiDesAb-O or DesAb-O at a 0.5, 1.0 and 2.0 molar stoichiometry. Now, in number of

paratopes this concentration is doubled in the DiDesAb-O relative to the DesAb-O (both containing one His6 tag per molecule). Therefore, the presumed gain in efficacy using a DiDesAb-O is most probably less pronounced as claimed.
In conclusion, there are doubts on the dimerisation construct, its performance/improvement is only qualitatively described and not quantitatively, and most experiments were already done for the monomeric DesAb-O as described in Aprile et al 2020 and Bigi, Napolitano et al , 2024.
A minor comment: some of the experiments were done with synthetic Aβ42, but many were performed on recombinant Aβ42, although the M&M does not describe how these recombinant target molecules were obtained a purified.

Reviewer #3

(Remarks to the Author)
The paper by Napolitano et al. showed that DiDesAb-O, which is a combination of two a dimeric DesAb-Os, improved the binding affinity to amyloidoligomers and attenuated induced neurotoxicity in cultured cells by synthetic amyloid oligomer and/or cerebrospinal fluid of AD patients. The results are very interesting and the manuscript is well written. However, there are some concerns before publications.

1) This manuscript clearly demonstrates that DiDesAb-O detects synthetic amyloid oligomers with high specificity. Howevwer, it is not clear whether DiDesAb-O can detect amyloid oligomers in AD patients. It is important for DiDesAb-O to recognize amyloid oligomers in AD patients. In this point of view, it is very interesting for DiDesAb-O to prevent neurotoxicity induced by cerebrospinal fluid of AD patients. Could you please employ Western blots using cerebrospinal fluid of AD patients and controls probed with DiDesAb-O and DesAb-Os? The results may provide us with a clue to identify the amyloid oligomer bodies present in the cerebrospinal fluid of AD patients to which DiDesAb-O binds.

2) Figure 7 showed that not only the cerebrospinal fluid from AD but also the cerebrospinal fluid from the control group significantly increased the intracellular calcium concentration, which was inhibited by DiDesAb-O. Does this mean that the cerebrospinal fluid from the control group also contains amyloid oligomers? Please discuss it in the section of Discussion.

Version 1:

Reviewer comments:

Reviewer #1

(Remarks to the Author)
I had already considered that this manuscript could be published as it stands. The changes made in this revision reinforce its scientific interest.

Reviewer #2

(Remarks to the Author)
The authors showed their willingness to meet the critics and comments raised by the reviewers.
However, their amendments are not really integrated into the text, it has just been added somewhere. For example the design of the DiDesAb-O is partially mentioned in M&M where no mention is made of the pre-linker also present in the spacer region between the 2 sdAbs. The complete sequence has to be found in the first paragraph of the Results section, where they refer to the supplementary material. Same argument for the descrioption of the recombinant Abeta. Why shoudl this be in the supplementary material and not in the M&M section of the body text. Moreover, it seems that the recombinant Abeta is fused with spider silk protein. Probably this is to have an increased expression and/or solubility. There is no mention that this tag is removed or how the protein is purified apart from solubilising (probably) inclusion bodies and centrifugations Therefore, I'm wondering how well this fusion peptide reflects the behaviour of synthetic or pure Abeta42? The details of the affinity measurement via SPR has to be found in the supplementary material. Here apparently the heterogeneous ADDL has been covalently linked on the CM5 chip up to 1000 RU which is an enormous amount (too much). Then low amounts of analyte are flown over the chip using the singel cycle kinetics. From the sensorgrams (A) it will be hard to measure the plateau values for each concentration. and also teh repetition experiment (3 runs) show that most likely the ligand is alos degraded/removed. The authors also didn't analyse teh k_off rate of the complex. Form the calculated KD value and the k_off rate, we can deduce the k_on rate as well. Knowledge of the kinetic rate constants would alos be very valuable as an identical KD can be reached by widely different k_on and k_off rates. Only the snesorgrams of the DesAb-O at the highest concentration can be used for KD measurement. Moreovere the calculations were performed under the estimation that it would be a 1:1 binding, which is clearly not the case. Daespite these shortcomings, we have a rough idea of the ratio of KD between the two ligands (sdAbs) and it seems that they differ 1000-fold. This is surprising, because in all other sdAb work we observe a 20 to 100 fold affinity increase upon generating tandem repeats of a monomeric sdAb. Therefore, it remains possible that the preferred target (i.e. teh target conformation with highest affinity for the ligand) for the monomer and the dimerised sdAb are different.

The Real time ELISA: The monomeric Abeta-(spidersilk, presumably) is put at 37°C and allowed to form gradually aggregates until fibrils. At 30min, 1 hr, 2 hrs and 22 hrs an aliquot is taken and coated in wells of microtiter plates at 4°C overnight. Presumably the aggregation/fiber formation is ongoing during this ON incubation. So the coated material does no longer reflects the state taken at the different time points. So here again, if your monomeric and dimeric sdAb binds preferentially to different aggregation forms, it will be difficult to draw any conclusions.

Furthermore, the TEM images indeed show that the A-beta fibers in presence of mono or dimeric sdAb differ from those without sdAb differ in morphology. However, the difference in fiber diameter is less clear. It is obvious that the dimeric sdAb leads to fibers with a jagged appearance. and possibly globular structures. But are these due to the Abeta or to the spider silk protein? it is never clear cut. So the conclusion (line 486) "reduction of fibril diameter is still questionable. Only an increased structural fragility for 'Abeta-spider silk' aggregation is evident.

Moreover, most experiments have been doen with microMolar amounts of the sdAb material. For the monomeric sdAb this means that the association constant is barely reached, whereas with the Dimeric sdAb it is 1000 fold excess over the association constant.

In summary, we are never sure of what we are looking at. The only thing that can be claimed is that the dimerised sdAb binds with much higher affinity than the monomeric sdAb and are therefore more suitable to bind and to exert an effect. This has however, been demonstrated multiple times with other systems.

Line 63 Position emission tomography or positron emission tomography????
Line 191: probably the authors used rabbit anti-mouse IgG conjugated to HRP not a "rabbit anti-mouse HRP conjugated secondary Ab"
Line 380, 381: is it figure S4A or Fig S5A?
Line 440: diameter instead of dimeter.

Reviewer #3

(Remarks to the Author)
According to reviewers' comments, most of the issues have been revised. My concern is that the raw data for Figure 7 was mixed up. If true, it shows very good results. However, I would like to leave it to the editor's discretion to approve this correction.

Open Access This Peer Review File is licensed under a Creative Commons Attribution 4.0 International License, which permits use, sharing, adaptation, distribution and reproduction in any medium or format, as long as you give appropriate credit to the original author(s) and the source, provide a link to the Creative Commons license, and indicate if changes were made.
In cases where reviewers are anonymous, credit should be given to 'Anonymous Referee' and the source.
The images or other third party material in this Peer Review File are included in the article's Creative Commons license, unless indicated otherwise in a credit line to the material. If material is not included in the article's Creative Commons license and your intended use is not permitted by statutory regulation or exceeds the permitted use, you will need to obtain permission directly from the copyright holder.
To view a copy of this license, visit https://creativecommons.org/licenses/by/4.0/

We sincerely thank the reviewers for their thorough and constructive feedback. In response, we have substantially strengthened the manuscript by performing new experiments, including affinity determination for both nanobody formats and additional dot blot assays with aggregation samples and patient-derived samples. Moreover, we have re-analized our data, as recommended. Below, we provide a detailed point-by-point response, which we trust fully addresses the reviewers' concerns.

Reviewer #1

This article is part of intensive research into Alzheimer's disease (AD), with a particular focus on soluble Aβ42 oligomers, identified as the most neurotoxic entities. The need for detection probes and therapeutic agents specific to these oligomers is crucial, given their pathogenic role and the difficulty of targeting them specifically.

The authors aim to improve the binding properties of an existing VHH or nanobody, DesAb-O, specific for Aβ42 oligomers. They designed a dimeric version, DiDesAb-O, to increase sensitivity and efficacy through an avidity effect. The design of dimeric variants has been widely used over the past decade, mainly in virology, to increase neutralising activity via an increase in avidity.

The authors have carried out an in-depth analysis of the dimeric variant: structure, effect on fibrils and prevention of toxicity. All these results are interesting but not really remarkable.

However, in the final section, the authors analysed the neutralising role of the dimer on the toxicity of oligomers present in the CSF of AD patients. They showed that the dimer is much more neutralising than the monomer.

This study is well documented and it is interesting to raise a dimer to improve the properties of the monomer against AD oligomers.

I consider it worthy of publication in Communications Biology

We thank the reviewer for the careful reading of our manuscript and for the very positive assessment of our work. We are particularly pleased that he/she found our approach of generating a dimeric version of DesAb-O (DiDesAb-O) to improve binding and neutralizing properties against $A\beta_{42}$ oligomers to be interesting and worthy of publication.

Reviewer #2

The authors started with their improved designed single domain antibody referred to as DesAb-O that binds to the AA stretch 29-36 of Aβ42. This DesAb-O (Aprile et al, 2020) had a relative weak affinity for Aβ42 oligomers and binds in a ratio of 1/5 (DesAb-O to Aβ42).

It is well established that the affinity of a weak recombinant binder can be approved by generating tandem repeats of this binder. Due to avidity effects such a dimerised binder will associate with its antigen (probably containing multiple epitopes) with a ≈100 fold lower KD value.

Hence the authors constructed a dimerised form of their DesAb-O joined by the standard (G4S)3 linkers and referred to this dimerised binder as DiDesAb-O. Consulting figure S1, providing the AA of the DiDesAb-O it appears that the construct also has a 36 amino acid leader sequence containing the His6 purification tag and downstream of the (G4S)3 linker a similar leader sequence has been introduced (but lacking the His6 tag). The reason to have this oligopeptide leader sequence or its origin are not mentioned. Thus what about the possible immunogenicity of these peptides? and what about the extravasation when such DiDesAb-O would be administered intravenously (see Debie et al, J. Control Release, 2020), and what about the BBB crossing? As long as in vitro experiments or on cell lines are performed you will not see any adverse effects

but in vivo (organisms) these linkers will certainly have a negative effect, and thus prevent any subsequent translational efforts.

We thank the reviewer for these important translational considerations. We would like to clarify that this pilot study is specifically aimed at determining whether dimerization of DesAb-O improves target engagement *in vitro*. The additional sequences visible in Figure S1, *i.e.*, the N-terminal leader containing the His_6 purification tag and the short sequence downstream of the $(G_4S)_3$ linker, are standard expression and purification elements used solely for recombinant production in a research setting. They are not intended to be part of any therapeutic candidate. The nanobody scaffold is already humanised to minimize immunogenic potential. Any future translational efforts would focus on removing all non-native leaders and tags, retaining only a minimal glycine–serine linker, and performing routine de-immunization and developability assessments. We do not investigate immunogenicity, extravasation, or blood-brain barrier delivery here as these issues are outside the scope of this in vitro proof-of-concept study; if advanced, the construct will be optimized for in vivo administration and evaluated through dedicated pharmacokinetics, biodistribution, immunogenicity, and brain delivery studies, in line with the considerations highlighted by Debie et al., J. Control Release, 2020.

The authors compared the effect of this DiDesAb-O relative to the DesAb-O for binding to Aβ42 conformers and the possible neutralisation of their toxicity. Strangely enough no attempts have been made to measure the apparent KD values or the binding stoichiometry.

We thank the reviewer for this valuable suggestion. In response, we have now performed a direct head-to-head comparison of DesAb-O and DiDesAb-O using surface plasmon resonance (SPR) under identical conditions (ADDLs immobilized at ~1000 RU; single-cycle kinetics at 37 °C). Our new data reported in the new Fig. S3E show that DiDesAb-O binds ADDLs with an apparent K_D of 5.0 ± 1.3 nM (left panel), while DesAb-O requires micromolar injections to yield a measurable response, resulting in an apparent K_D of 4.9 ± 3.6 μM (right panel), approximately a 10^3-fold affinity advantage for the dimer. The K_D value measured for DesAb-O is higher than our previous solution ITC estimate (~0.4 μM; Aprile et al., PNAS, 2020), which is reasonable considering that different stabilized oligomer preparations and experimental setups were used.

These new results are now reported in the revised manuscript (Results section, pages 11-12) and in the supplemental material (new Figures S3E and S4, new Table S1; Supplementary methods section, page 2-3) and confirm that dimerization markedly enhances binding affinity to Aβ42 oligomers.

The results seem to confirm that the DiDesAb-O performs better than the monomeric DesAb-O, as expected. However, one might wonder whether these statements are justified. The comparison were made using a fixed Aβ42 concentration (1 μM) and adding DiDesAb-O or DesAb-O at a 0.5, 1.0 and 2.0 molar stoichiometry. Now, in number of paratopes this concentration is doubled in the DiDesAb-O relative to the DesAb-O (both containing one His6 tag per molecule). Therefore, the presumed gain in efficacy using a DiDesAb-O is most probably less pronounced as claimed.

We thank the reviewer for this important observation. In response, we have re-analyzed our data to control for paratope equivalence by comparing DiDesAb-O at each stoichiometry to DesAb-O at twice the concentration. This revised analysis confirmed that DiDeAb-O remains significantly more effective than DesAb-O at matched binding-site concentrations. As shown in Fig. 5 (membrane-binding assay), DiDesAb-O inhibited Aβ42 oligomer binding to cellular membranes more effectively than paratope-equivalent concentrations of DesAb-O. (Student's t-test, $P < 0.01$). Moreover, in Fig. 6 DiDesAb-O provided superior protection against Aβ42-induced calcium dysregulation and mitochondrial dysfunction (MTT assay) even after paratope normalization, with statistically significant differences (P values reported in figure legends). These findings support that the enhanced efficacy of DiDesAb-O is not solely due to the doubled number of paratopes, but likely reflects avidity and/or cooperative binding effects. The shared His_6 tag on both constructs cannot account for the observed differences.

We have revised the legends of Figure 5 and 6 accordingly to explicitly state that some comparisons were performed at equivalent paratope concentrations and to report the corresponding statistical analyses.

In conclusion, there are doubts on the dimerisation construct, its performance/improvement is only qualitatively described and not quantitatively, and most experiments were already done for the monomeric DesAb-O as described in Aprile et al 2020 and Bigi, Napolitano et al , 2024.

We thank the reviewer for these remarks. As described above, we have strengthened the quantitative assessment of DiDesAb-O efficacy by performing a new head-to-head SPR analysis directly comparing DesAb-O and DiDesAb-O under identical conditions. These data provide apparent K_D values and confirm a ~10^3-fold affinity gain for DiDesAb-O (see the supplemental material: new Figures S3E and S4, new Table S1; Method section, pages 2-3. See the revised manuscript: Results section, pages 11-12). Furthermore, we have re-analyzed all functional assays at paratope-equivalent concentrations, demonstrating that DiDesAb-O retains significantly superior efficacy compared with DesAb-O (P values now reported in figure captions). This addresses the concern that the apparent gain might simply reflect a doubled paratope number. We have revised the legends of Figure 5 and 6 accordingly to explicitly state that some comparisons were performed at equivalent paratope concentrations and to report the corresponding statistical analyses. Together, these new quantitative data clearly support that dimerization confers a genuine increase in affinity and neuroprotective potency beyond what could be expected from paratope counting alone, thus providing an advance over previously published monomeric data.

A minor comment: some of the experiments were done with synthetic Aβ42, but many were performed on recombinant Aβ42, although the M&M does not describe how these recombinant target molecules were obtained and purified.

We have now added a few details about the purification procedure for recombinant $A\beta_{42}$ in the Supplementary Materials and Methods section (page 1).

Reviewer #3

The paper by Napolitano et al. showed that DiDesAb-O, which is a combination of two a dimeric DesAb-Os, improved the binding affinity to amyloid oligomers and attenuated induced neurotoxicity in cultured cells by synthetic amyloid oligomer and/or cerebrospinal fluid of AD patients. The results are very interesting and the manuscript is well written. However, there are some concerns before publications.

1) This manuscript clearly demonstrates that DiDesAb-O detects synthetic amyloid oligomers with high specificity. However, it is not clear whether DiDesAb-O can detect amyloid oligomers in AD patients. It is important for DiDesAb-O to recognize amyloid oligomers in AD patients. In this point of view, it is very interesting for DiDesAb-O to prevent neurotoxicity induced by cerebrospinal fluid of AD patients. Could you please employ Western blots using cerebrospinal fluid of AD patients and controls probed with DiDesAb-O and DesAb-Os? The results may provide us with a clue to identify the amyloid oligomer bodies present in the cerebrospinal fluid of AD patients to which DiDesAb-O binds.

We thank the reviewer for this insightful suggestion. To directly address this point, we employed our validated sandwich dot blot assay (Bigi, Napolitano et al., 2024) to compare the ability of DiDesAb-O and DesAb-O to detect both on-pathway $A\beta_{42}$ oligomers in a reconstituted system *in vitro* and species present in human CSF. As reported in the new Fig. 7A, DiDesAb-O showed maximal reactivity at 8 h of in vitro aggregation, a minor signal at 0 h, and a low signal at 24 h, consistent with specific recognition of on-pathway oligomers; DesAb-O also peaked at 8 h but retained a clear 0 h signal, indicative of partial monomer binding (Aprile et al., 2020; Bigi, Napolitano et al., 2024). Notably, DiDesAb-O produced a strong signal with CSFs from AD patients and only a weak signal with age-matched controls, supporting selective detection of pathological $A\beta_{42}$ oligomers in patient-derived samples. We have revised the manuscript accordingly (Abstract; Introduction section, page 2; new Fig. 7A; Materials and Methods section, page 7; Results section, page 18; Discussion section, page 20)

2) Figure 7 showed that not only the cerebrospinal fluid from AD but also the cerebrospinal fluid from the control group significantly increased the intracellular calcium concentration, which was inhibited by DiDesAb-O. Does this mean that the cerebrospinal fluid from the control group also contains amyloid oligomers? Please discuss it in the section of Discussion.

We thank the reviewer for drawing our attention to this important point. Upon re-examination of the raw data, we discovered that one AD CSF sample and one control CSF sample had been inadvertently swapped, which led to the false impression that control CSF significantly increased intracellular Ca^{2+}. After correcting the sample labels and re-analyzing the data, we confirm that control CSF does not induce a significant increase in intracellular Ca^{2+}, whereas AD CSF does, and this effect is robustly prevented by DiDesAb-O.

Accordingly, Figure 7, its legend, and the Results section (page 18 for the Results section, and page 34 for the figure legend) have been corrected to explicitly clarify that control CSF does not elicit toxic Ca^{2+} responses under these conditions.

For transparency, we also report below the old and new individual values for each CSF sample. The swapped values are highlighted in orange (CSF 2) and red (CSF 3).

**Table - new values**				**Table - old values**			
**CSF AD**		**CSF cntrl**		**CSF AD**		**CSF cntrl**	
**CSF 1**	220,3	**CSF 1**	119,4	**CSF 1**	220,3	**CSF 1**	119,4
**CSF 2**	196,6	**CSF 2**	144,1	**CSF 2**	196,6	**CSF 2**	185,8
**CSF 3**	185,8	**CSF 3**	127,9	**CSF 3**	144,1	**CSF 3**	127,9
**CSF 4**	201, 24	**CSF 4**	114,8	**CSF 4**	201,2	**CSF 4**	114,8
**Average**	**200,9**	**Average**	**126,55**	**Average**	**190,55**	**Average**	**136,975**

Furthermore, in line with our commitment to data integrity, we conducted a thorough and critical re-examination of our entire dataset. During this process, we identified a minor technical error in the data processing related to the experiment assessing Aβ42 oligomers interacting with neuronal cells and penetrating into the cytosol upon treatment with DiDesAb-O. To ensure the highest level of rigor and reproducibility, we corrected the data processing pipeline and performed a critical reassessment of the experimental design. The dataset was then re-analyzed using a one-tailed Student's t-test, which provides a more focused and appropriate statistical comparison for this type of analysis. Importantly, the re-analysis fully supports and further validates our original conclusion that DiDesAb-O exhibits an enhanced ability to recognize Aβ42 bound to the cellular membrane at lower concentrations compared to DesAb-O. Accordingly, the Results section (page 16) the Figure 4 and legend have been corrected.
